# Location and condition based reconstruction of colon cancer microbiome from human RNA sequencing data

**DOI:** 10.1186/s13073-023-01180-9

**Published:** 2023-05-02

**Authors:** Gaia Sambruni, Angeli D. Macandog, Jakob Wirbel, Danilo Cagnina, Carlotta Catozzi, Tiziano Dallavilla, Francesca Borgo, Nicola Fazio, Uberto Fumagalli-Romario, Wanda L. Petz, Teresa Manzo, Simona P. Ravenda, Georg Zeller, Luigi Nezi, Martin H. Schaefer

**Affiliations:** 1 Department of Experimental Oncology, European Institute of Oncology-IRCCS, Milano, Italy; 2 Structural and Computational Biology Unit, European Molecular Biology Laboratory, Heidelberg, Germany; 3 Center for Omics Sciences, IRCCS San Raffaele Institute, Milano, Italy; 4 Division of Gastrointestinal Medical Oncology and Neuroendocrine Tumors, European Institute of Oncology-IRCCS, Milano, Italy; 5 Digestive Surgery, European Institute of Oncology-IRCCS, Milano, Italy

**Keywords:** Tumour microbiome, RNA-Seq data deconvolution, Microbe-tumour interaction, Microbiome biomarker

## Abstract

**Background:**

The association between microbes and cancer has been reported repeatedly; however, it is not clear if molecular tumour properties are connected to specific microbial colonisation patterns. This is due mainly to the current technical and analytical strategy limitations to characterise tumour-associated bacteria.

**Methods:**

Here, we propose an approach to detect bacterial signals in human RNA sequencing data and associate them with the clinical and molecular properties of the tumours. The method was tested on public datasets from The Cancer Genome Atlas, and its accuracy was assessed on a new cohort of colorectal cancer patients.

**Results:**

Our analysis shows that intratumoural microbiome composition is correlated with survival, anatomic location, microsatellite instability, consensus molecular subtype and immune cell infiltration in colon tumours. In particular, we find *Faecalibacterium prausnitzii*, *Coprococcus comes*, *Bacteroides* spp., *Fusobacterium* spp. and *Clostridium* spp. to be strongly associated with tumour properties.

**Conclusions:**

We implemented an approach to concurrently analyse clinical and molecular properties of the tumour as well as the composition of the associated microbiome. Our results may improve patient stratification and pave the path for mechanistic studies on microbiota-tumour crosstalk.

**Supplementary Information:**

The online version contains supplementary material available at 10.1186/s13073-023-01180-9.

## Background

Tumours are evolutionary systems and natural selection operates on their genomes, facilitating adaptation to the environment [1]. Therefore, the composition of the microenvironment has a profound impact on the selective forces shaping the tumour genome and may lead to distinct molecular subtypes. In this regard, the tumour microbiota is emerging as a significant determinant [2, 3], as demonstrated by the association of gastrointestinal dysbiosis with colorectal cancer [4, 5] and the impact of microbiota on tumour initiation, progression and therapy response [6]. However, to date only a few bacterial species have been shown to have oncogenic or cancer-supportive capabilities [7], including *Helicobacter pylori* in gastric cancer [8] and *Fusobacterium nucleatum* and colibactin producing *Escherichia coli* in colon cancer [9, 10]. The interactions of bacteria with tumours engage specific metabolic activities along with physical contact and can modulate the host immune system [11, 12]. Nevertheless, if and how bacteria contribute to shaping the molecular properties of the tumour and influence the clinical outcome is still poorly understood [13–17]. Cancer is a highly heterogeneous disease and the detection of subtypes and molecular characteristics of patients drove the development of personalised treatment strategies [18]. For personalised medicine, the microbiome plays a minor role so far. To change this, the first step would be to understand how the microbiome varies between subtypes and is associated with specific properties of the tumour. Addressing these points is currently challenging as it requires simultaneous characterisation of the microbiome and tumour properties of a large number of patient samples.

Recent studies have begun to explore the possibility of extrapolating information on tumour-associated bacteria from widely available human sequencing data, e.g. from whole exome sequencing (WXS) [19–26] or RNA sequencing (RNA-Seq) [20, 23–25, 27, 28]. Most of these studies focused on the differences between cancer types or tumour versus control tissue [20–24, 26, 28]. Recent studies suggested a link between the presence of specific bacteria in tumour samples and clinical properties of the tumours [21, 22, 25–27]. However, how much these associations could be influenced by batch effects and contamination is still under debate [19–22, 27] and a systematic study of the link between molecular, clinical and prognostic properties of tumours with their microbiome is still missing.

**Fig. 1:**
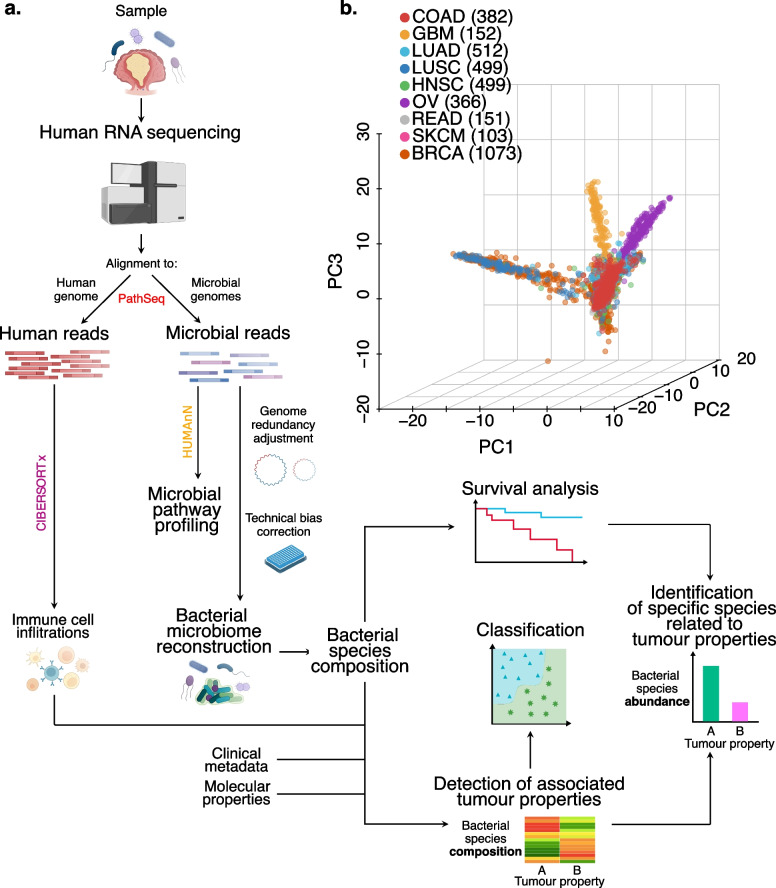
General overview on bacteria reconstructed microbiomes. **a** Summary of the microbial reconstruction workflow to detect clinical and molecular associations with bacteria. Each RNA sequencing BAM file was analysed by this workflow: after removing the human sequences, the rest of the reads were aligned to a set of microbial genomes from the National Center for Biotechnology Information (NCBI) and assigned to one or more species. The last step to reconstruct the microbiome of the samples involves a batch effect detection that identifies the influence of available technical properties on the reconstructed microbiome composition. Microbiome profiles are then corrected accordingly for the most relevant batch effects. Finally, the corrected microbiome profiles were tested for associations with clinical and molecular properties of the tumour, survival analysis and property classification. For the properties associated with microbiome composition, the bacterial composition underwent a property classification approach, while specific bacterial species were detected as linked to the property levels and the prognosis. Colon adenocarcinoma (COAD) bacterial reads were pooled into left and right-sided, CMS1 and CMSs pooled and mutation burden high and low and underwent pathway profiling to compare metabolic differences between the sides of the colon, CMSs and mutational burden levels, respectively. **b** Principal component analysis on all the reconstructed bacterial microbiomes of the cancer types analysed. GBM, glioblastoma multiforme; LUAD, lung adenocarcinoma; LUSC, lung squamous cell carcinoma; HNSC, head and neck squamous cell carcinoma; OV, ovarian serous cystadenocarcinoma; READ, rectum adenocarcinoma; SKCM, skin cutaneous melanoma; BRCA, breast invasive carcinoma. Number of samples analysed in brackets

We implemented a computational workflow [29] to extract microbial reads from human RNA-Seq data, identify and eliminate experimental contamination and quantify the associations between properties of the tumour and the species-level microbiome composition (Fig. 1a). We applied our workflow to colon, lung, brain, head and neck, ovary, skin and breast tumour samples from The Cancer Genome Atlas (TCGA) to reconstruct the tumour-specific microbiome. Subsequently, the accuracy of this workflow in inferring microbiome composition was validated in a novel cohort of colon cancer patients in which we simultaneously sequenced the tumour and quantified bacterial abundances by two independent approaches. Our results indicate strong associations between the bacterial composition and molecular, clinical and prognostic properties of the tumour and highlight specific bacterial species potentially associated with them. Finally, we explored associations with the immune compartment and bacterial metabolic peculiarities in the left and right colon.

## Methods

### Samples

After obtaining the required permission, we downloaded the RNA-Seq data from 3737 primary tumour and 318 solid tissue normal control samples from NCI Genomic Data Commons (GDC)/TCGA. The non-malignant samples were taken during the biopsy of cancer patients from an adjacent area from the tumour site. The analysed samples belonged to colon adenocarcinoma (COAD [30], 382 primary tumour, 39 solid tissue normal), glioblastoma multiforme (GBM [31], 152 primary tumour, 5 solid tissue normal), lung adenocarcinoma (LUAD [32], 512 primary tumour, 59 solid tissue normal), lung squamous cell carcinoma (LUSC [33], 499 primary tumour, 49 solid tissue normal), head and neck squamous cell carcinoma (HNSC [34], 499 primary tumour, 44 solid tissue normal), ovarian serous cystadenocarcinoma (OV [35], 366 primary tumour), rectum adenocarcinoma (READ [30], 151 primary tumour, 9 solid tissue normal), skin cutaneous melanoma (SKCM [36], 103 primary tumour, 1 solid tissue normal) and breast invasive carcinoma (BRCA [37], 1073 primary tumour, 112 solid tissue normal) patients. To avoid any possible intra-study bias caused by different sample preparations, we only considered samples analysed by the Illumina Truseq method (in COAD, LUAD and BRCA) and AllPrep RNA extraction (in OV) and removed the cases with duplicated samples (GBM and LUSC); the information about the preparation steps was downloaded from the GDC Legacy Archive [38].

We additionally enrolled a cohort of 30 resectable colon cancer patients prospectively from European Institute of Oncology (IEO) hospital. The local Ethics Committee approved the study and each patient was asked to sign an informed consent. We obtained from each patient a tissue sample of the tumour and non-tumour adjacent region (at 2 and 10 cm from the border of the pathologist-assessed neoplastic lesion). Samples underwent RNA-Seq (90 samples), ribosomal RNA 16S gene (16S) sequencing (61 samples) and bacterial fluorescence in situ hybridisation (FISH, 10 samples). Two samples with low amounts of RNA-Seq bacterial reads (less than 300 reads) were removed from the final analyses.

### RNA extraction, sequencing and analysis

RNA was extracted from flash-frozen tissues using the AllPrep DNA/RNA kit (Qiagen) following manufacturer recommendations. One hundred nanograms was used for RNA library preparation using the Illumina Truseq or Stranded Total RNA Prep Ligation with Ribo-Zero Plus kit (Illumina). In brief, after depleting rRNA, the RNA was fragmented at 94° C for 2 min. After retrotranscription and anchor ligation, the library was amplified (13 cycles). Sample quality and quantity were checked again by Bioanalyser and Qubit, respectively and then sequenced (50 base pair paired-end reads) by Illumina NovaSeq 6000.

Intending to reduce as much as possible the differences in the bioinformatic approaches used to analyse GDC/TCGA and the IEO cohort samples, we processed the IEO cohort samples with the same tools and the same parameters described by TCGA. We ran the STAR aligner (GRCh38) on the IEO cluster with the same parameters described in TCGA documentation (https://docs.gdc.cancer.gov/Data/Bioinformatics_Pipelines/Expression_mRNA_Pipeline/).

### FISH

Carnoy’s fixed and paraffin-embedded tumour tissues were processed for FISH following a modified version of the previously published protocol [39]. Briefly, after deparaffination and rehydratation, tissue slides were incubated for 3 h in the hybridization buffer (with a specific temperature and amount of formamide depending on each probe) with FISH probes targeting most of bacteria universally (EUB probe) in combination with probes targeting one of the following species specifically: *Akkermansia muciniphila* and *Faecalibacterium prausnitzii* (Additional file 1: Table S1). Then, slides were washed, incubated with 4’,6-diamidino-2-phenylindole (DAPI) for nuclei staining and mounted. Image acquisition was performed using an SP8 confocal microscope (Leica) at × 63 magnification. Example images are shown in Additional file 1: Fig. S1a-b.

The comparison between RNA-Seq and FISH values was done using Spearman correlation for each analysed species. Data were calculated by normalising the bacterial counts from FISH images by the number of EUB positive signals and total cells (DAPI) present in the images.

### DNA extraction, 16S sequencing and analysis

We isolated a mucosal scrap from each tissue sample by gently scraping the mucosa with 1 ml of phosphate buffered saline (PBS) solution. Of this, 500 µl was used to extract DNA using the DNA Power Soil Pro Isolation kit (Qiagen). DNA was quantified by Qubit, and the quality was assessed by Nanodrop. The amplification and sequencing of the 16S V3–V4 regions were performed following the 16S Metagenomic Sequencing Library Preparation protocol [40]. Briefly, the first PCR (25 cycles) was performed using the 16S V3–V4 primers under the manufacturer’s instructions. Both forward and reverse primers (Additional file 1: Table S1) were composed of Illumina overhang adapter sequences and the specific 16S sequences of primers. Then, the second PCR (8 cycles) was carried out to attach dual indices and Illumina sequencing adapters using the Nextera XT Index Kit. After pooling, DNA quality and quantity were checked by Bioanalyser and Qubit and run on a MiSeq flowcell (Illumina).

For validation of RNA-Seq data, the IEO cohort samples underwent 16S V3–V4 amplicon sequencing. The 16S sequences were analysed using qiime2 [41]. Raw count tables were produced using q2-dada2 [42] with truncation length parameters set to the primer length. Taxonomic profiling was done by trimming whole-gene 16S sequences from the SILVA 132 database [43] by the flanking region of the V3–V4 primers. The trimmed SILVA sequences were trained with the q2-classifier skclassify plug-in [44], after which the trained classifier was run on the representative sequences output of DADA2 [42]. The majority of the sequences were resolved to genus level, so the output taxonomy table was collapsed to genus level and transformed into relative frequencies for further analysis.

The comparison between RNA-Seq and 16S was made by subsetting the datasets to include only the intersecting genera between the two and then doing the Spearman correlation test on each genus. To understand how rare, low-abundance taxa (with many zero abundance values) are affecting the correlation, prevalence filtering was applied to both datasets, where genera that are present (i.e. bacterial relative abundance > 0) above a percentage threshold of number of samples in both datasets were kept (0%, 10%, 20%, 30%, 40%, 50% and 60%). The distribution of the Spearman coefficients across prevalence filter thresholds was visualised with a density plot, using one-sample Wilcoxon to test at each cut-off whether the median of the distribution is greater than zero (Additional file 1: Fig. S1c).

### Tumour properties

Among the clinical information of samples provided by TCGA, we selected those that are lowly redundant, are available for most of the patients and are considered clinically relevant. In particular, we considered gender, body mass index (BMI), stage, history of other malignancy, side, age at initial pathological diagnosis, history of colon polyps and percentage of normal cells. Among all the properties reported by TCGA, we also considered technical properties from which we assumed that they could potentially affect our results. Among the residual properties, we decided to select the ones that could be associated with the microbiota composition. To expand our analysis of clinical properties of the tumours, we took advantage of some previously published analyses on TCGA cohorts: we considered microsatellite instability (MSI) level [45] (as suggested by the authors, we classified as high MSI those samples with a MANTIS score > 0.4 and with a low MSI the ones with MANTIS score < or equal to 0.4), the CpG methylation phenotype (CIMP) status [46], the consensus molecular subtype (CMS) classification (determined from the tumour gene expression profile with the CMSclassifier R package [47]) and the stemness value [48].

We also considered two molecular properties of tumours: the aneuploidy status [49] and the driver gene mutation status. We quantified the status of the most frequently mutated genes in colorectal cancer and the other cancer types [50] as the total number of mutations found in each TCGA sample using the GDC database collection [51]. We considered a gene mutated if it carries any type of non-silent mutation (silent mutations: silent, 5'flank, RNA, intron, 3'flank).

Finally, we inferred immune cell infiltration by running CIBERSORTx [52] from their web page on transcript per million (TPM) gene expression quantification with the default signature matrix LM22, B-mode batch correction activated, with 1000 permutations in both absolute and relative mode. We considered only the significant (*p* < 0.05) immune estimates. TPM were calculated from fragments per kilobase million (FPKM) tables from the GDC by dividing each FPKM value with the sum of the FPKM values of that sample and then multiplied by 1 million. Ensembl IDs were converted to HUGO gene names using the annotation version v22. We tested continuous properties with a specific test (i.e. Spearman correlation test), but if needed (i.e. for the independence test), we converted the continuous variables to discrete ones by binning the properties. To this end, we applied an approach to automatically find the best break points: if the frequency of zero values is over 30%, we considered the presence or absence (anything above zero considered as presence); if the distribution was normal (Shapiro test) or the distribution was bimodal (is.bimodal function from LaplacesDemon R package), we defined low and high values taking the mean or the lowest value between the two peaks as break, respectively; and if none of the previous conditions were satisfied, we binned the values by quartiles (low, medium–low, medium–high and high levels).

### Microbiome reconstruction workflow

Our computational workflow [29] consisted of five steps:

1. Microbial read extraction: we applied PathSeq from the Genome Analysis Toolkit [53] using the provided reference genomes prepared on 12/04/2017 (human: GRCh38). We ran the tool PathSeq [54] with default parameters and for each bacterial species we used the “score” values from the PathSeq output matrix to evaluate bacterial abundances: they take into account that species share homologous genomic regions. A read that maps to a common region cannot be assigned to only one taxon, so PathSeq provides a “weighted count” of the number of reads that map to the reference genome of the taxon considered. Considering taxon *t*, if a read maps only to the genome of the taxon *t*, it has a value of 1; if it does not map, it has a value of 0; and if the read maps to more than one genome (to a common region), it has a value of 1/(number of genomes to which the read maps). The bacterial score of the taxon *t* is the sum of the values from all the reads.
2. Genome redundancy adjustment: the reconstruction of the microbiome from human RNA-Seq with PathSeq can be affected by several problems: human samples can undergo contamination at different stages of processing (from surgery to sequencing) [23]. The detection of bacterial species can also be affected by the wrong identification of species due to technical reasons: technical sequencing errors (usually discarded in human analyses) can randomly map to bacterial genomes and the presence of common sequences shared by two (or even more) different species can alter the quantification or wrongly detect bacteria which are not present. To avoid taking into account the bacterial scores of non-detected species that share genomic regions with the real sample-derived ones, we only considered the bacterial scores of those species with at least one unambiguously mapping read. The bacterial score values were then intra-sample normalised so that all bacterial species scores sum up to 100 as a measure of bacterial relative abundance scaled to percentages.
3. Batch effect detection and correction: to detect the major technical batch affecting the bacterial composition of each cancer type, we measured the Euclidean distances of samples in the first six principal components (PCs) of the principal component analysis (PCA, collectively explaining more than 10% of the variability) of each cancer type. We compared the distributions of these distances of samples belonging to the same level of the technical property to the distances of samples belonging to different levels of that property by the Wilcoxon test. For example, we compared the distribution of the distances between the samples belonging to the same 96-well plate identifier (plate ID) to the distances between samples from different plate IDs. The technical property showing the lowest *p* value was considered the major batch effect in the analysed cancer type. For all the cancer types analysed, we determined that the plate ID is the most important batch effect, except for GBM samples in which no clear technical batch effects were found. In the IEO cohort, the dominant detected technical batch was the sequencing run. In the PCA, we noticed a separation between samples sequenced with a different read length in COAD and READ tissues (Additional file 1: Fig. S2a). In fact, read length was the second-most influential factor identified by our batch effect detection approach. Correcting for the plate IDs also reduced this read length effect (Additional file 1: Fig. S2b), since it is strongly associated with the plate IDs (Additional file 1: Fig. S2c). As major confirmation, we tested the clinical property associations with tumour COAD samples batch corrected for sequencing read length and we got similar results to the ones obtained correcting for plate IDs (Additional file 1: Fig. S2d-e, 3a). We also tested the clinical property association to the subset of tumour COAD samples with 48 bp-read length or 76-read length and observed that some of the associations still held (Additional file 1: Fig. S3b-c).
  To correct for the identified batch effect, the reconstructed bacterial microbiome relative abundances were scaled and log-transformed. After that, we applied the ComBat function from the sva package in R [55], controlling for the known batch covariate (the plate IDs or the sequencing run). Since some cancer types have few samples per plate, we pooled the plates with a low number of samples: plates were pooled if they had less than 10% of the total number of samples (frac), if frac > 5, frac was set to 5.
4. Microbiome composition PCA: to investigate the differences between the whole reconstructed microbiota of samples with PCA, we applied the prcomp function from the stats R package. Before applying the method, we removed the species with zero bacterial relative abundances in all the samples analysed. After this, we selected the 1000 species with the highest standard deviation values. The presence of outliers in the PCA can alter the results, so we measured the Euclidean distances between samples and, if one sample was the most distant to 95% of the other samples (or more), it was considered an outlier and removed. After removing an outlier, we reran the outlier identification method to identify and remove further outliers until no further ones could be detected.
5. Tumour property association with microbiome composition: to test the specific association between the PCs and the tumour properties, we ran the PCA and compared the different distributions of the PC coordinates with the subgroups we were analysing with the Wilcoxon or Kruskal–Wallis test. We also tested the correlation between PC coordinates and the tumour properties values with the Spearman correlation test. We considered the first six PCs since they can explain more than 10% of the total variability of the reconstructed microbiome for all the cancer types tested. For survival analysis, we considered as top PC-contributing species (200 species) with the highest absolute loading values of each PC of the PCA (Additional file 2: Table S2).

Since there are different ways to deal with reads mapping to sequence-redundant regions of bacterial genomes, we wanted to understand if these different ways to estimate bacterial signals could affect our results. To this end, we tested if different COAD bacterial abundance estimations could detect associations not previously found with the here described approach (steps 1 and 2). We applied the same workflow considering only uniquely mapping reads per species (i.e. the unambiguous reads) or using the sum of all the mapping reads, both unambiguous reads and reads mapping to redundant regions (i.e. the ambiguous reads). We detected and corrected for the strongest batch effect (i.e. the sequencing plate, as identified with our current approach) and tested for significant associations between the first six PCs of the reconstructed microbiome PCA and tumour properties. All the approaches found side, MSI, CIMP and aneuploidy status associated with the bacterial compositions of samples (Additional file 1: Fig. S3d), while the relaxed one (including ambiguous reads) detected also the association with the percentage of normal cells that was not previously identified.

To compare the variations of bacterial abundances of species related to specific properties of the tumour (e.g. the abundances in the left versus the right side of the colon in the primary tumour), we used the generalised log fold change, as described in Wirbel et al. [56].

### LASSO regression model

In order to test if the bacterial composition could serve as potential biomarker for clinical tumour properties, we trained a least absolute shrinkage and selection operator (LASSO) logistic regression machine learning model [57] to distinguish stage, MSI status, CMS and the percentage of normal cells across all COAD samples. To have a binary classification problem, the tumour properties were adjusted to obtain only two classes, when needed: the stage information was split into early (stages I and II) and late (stages III and IV) stage; for each CMS, a new label was created in which every CMS was grouped against the rest of the CMSs (e.g. CMS1 versus all other CMSs); finally, the percentage of normal cells were split by low (zero value) and high (over 10%). We selected 500 bacteria with the highest standard deviation (SD) on which we then trained a LASSO regression model with the SIAMCAT package in R [58], using a 10 times repeated tenfold cross-validation strategy. Given the repeated cross-validation, there are multiple predictions for each sample (whenever it was used as a test sample during a single round of cross-validation). We therefore averaged all predictions across the cross-validation repeats to get a single prediction per sample, which was then used to assess the accuracy of the model. For classification of non-malignant versus tumour samples, we selected 200 bacteria with the highest SD and grouped the samples by Patient ID during cross-validation, since we used only paired samples.

### Pathway analysis

To compare the pathways enriched in the tumour colon properties (e.g. the two sides of the colon), we pooled together the PathSeq output BAM files of the primary tumour samples from the same sublevel (e.g. from the left and the right sections). We then analysed these pooled reads with HUMAnN 3.0 [59] and, as suggested by the authors of this tool, we normalised the pathway abundances to copies per million (CPM). We filtered out the low-abundance pathways (abundance below the first quartile, 30.49 CPM on the left, 27.02 CPM on the right, 26.74 CPM in CMS1, 31.27 CPM in pooled CMSs, 26.18 CPM in high mutation burden and 25.36 CPM in low mutation burden) and then we considered the pathways showing at least one third higher abundance than the other sublevel (e.g. sides of the colon) as differentially active.

We applied bootstrapping to estimate the significance of our observations: we randomly picked one third of the samples from each sublevel in 50 independent permutations and applied HUMAnN as described above to obtain pathway distributions of the sublevels (e.g. in left and right). The distributions of the previously identified pathways were compared with the Wilcoxon test and a false discovery rate (FDR) multiple-test corrected *q* value < 0.2.

### Survival analysis

The cBioPortal for Cancer Genomics disease-free survival (DFS) and overall survival (OS) data were downloaded from cBioPortal [60, 61]. To measure the association between survival and the microbiome composition, we applied Cox proportional-hazard models with the coxph function of the survival R package. First, we ran univariate Cox models on the top six PC coordinates separately and selected the significant ones. To exclude the possible impact on survival due to clinical properties associated with PCs, we tested the selected PC coordinates together with their associated properties in a multivariate model and checked whether PCs remained significant. To take into account properties with different scales, we scaled continuous properties to be in the range 0–1. We further validated our results by running the Kaplan–Meier analysis on PC coordinates that are not confounded by associated properties (in this case, we used original PC coordinates). To stratify patients into “high” and “low” groups, maximally selected rank statistics were adopted. To detect which bacterium is associated with the relapse probability, we applied univariate Cox analysis to the batch corrected values of the first 100 bacteria with the highest loadings of the PCs associated with relapse probability (PC4). We then multiple-test correct the *p* value of the Wald test, selecting for *q* < 0.2 species.

### Filter criteria on bacteria

We applied two different approaches to select the species of interest:

1. High-confidence set of species: given the high number of bacterial species detected in the cancer types analysed, we defined three filters to remove the low-present, batch-affected bacteria and select the cancer type–specific ones. To remove the bacteria whose distribution is affected by the dominant batch effect of the cancer type, we applied the Wilcoxon test to their relative abundances and removed the bacteria with FDR multiple-test corrected *q* value < 0.1. To filter out the low prevalent bacteria, we selected those bacteria detected in at least 10% of the samples of the cancer type of interest. To select cancer type–specific bacteria, we finally selected those bacteria showing a higher mean in the cancer type analysed than in the other types.
2. Colon–specific set of species: we screened the bacteria by applying the presence and cancer type-specificity filters described above. After that, in order to test for differentially abundant species between different levels of properties, we applied a non-parametric Mann–Whitney test: the independence_test function as implemented in the R package coin [62]. To consider the batch effect present in the samples, we applied the independence_test blocking for the property we considered the dominant technical batch. Finally, we considered bacteria statistically significantly associated with a property, if their multiple-testing corrected *q* value (FDR method) was below 0.1.

## Results

### Microbiome reconstruction from RNA-Seq data of different cancer types

A computational workflow was implemented (Fig. 1a) to reconstruct the microbiome by extracting bacterial reads from RNA-Seq data [54], detecting and correcting for contaminants and batch effects and summarising global microbiome composition trends using dimensionality reduction (see “Methods”). We started by reconstructing the microbiome from nine TCGA RNA-Seq studies on epithelial tumours. These studies originate from tissues strongly exposed to microbiota, namely colorectal, head and neck, skin, lung and breast epithelial tissues; additionally, we analysed ovary, where exposure to microbiome is under debate [63] and brain tissue, which is largely sheltered from microbes in contrast with epithelial cancers (in total 3737 samples). This analysis yielded 59,592,060 bacterial reads (0.02% of the total reads; Additional file 1: Fig. S4a) mapping to 11,961 bacterial species and, surprisingly, detected bacterial signals in all the cancer types analysed, including brain tumours (GBM). Next, we aimed to remove those bacterial reads that might originate from contamination and bacteria only supported by ambiguous genomic regions. To minimise these effects, first, we included only the bacteria detected by at least one read mapping to a non-redundant region (18,236,650 unambiguous reads). Then, we used a bacterial score that weights the reads by the number of genomes they map to (based on 39,081,191 reads associated with 10,910 bacterial species; Additional file 1: Fig. S4b,c) (see “Methods”). Bacterial scores were then intra-sample normalised to obtain bacterial relative abundances. With this approach, we reduced the number of bacterial species detected per sample and recovered a significant fraction of reads shared by multiple genomes that would otherwise be discarded. We next established a step in the workflow to computationally correct the technical variation affecting the reconstructed microbiome (see “Methods” for details): to compare samples from different cancer types, we applied a PCA on the species of the bacterial microbiome showing the highest variability between samples (using the 1000 bacteria with the SD) (Fig. 1b). This approach revealed that the reconstructed microbiome clustered by cancer type, suggesting cancer type–specific bacterial composition. However, when we analysed each cancer type, technical factors were also critical for the clustering, with plate ID emerging as the strongest contributor. After correcting for these batch effects (see “Methods”), differences between the reconstructed microbiomes were only minimally affected by technical factors (Additional file 1: Fig. S4d), suggesting that our approach can quantify the presence of bacterial reads on a broad number of samples while controlling for sources of unwanted technical variation and noise in the data.

### Comparison with experimental detection approaches to characterise the microbiome

**Fig. 2:**
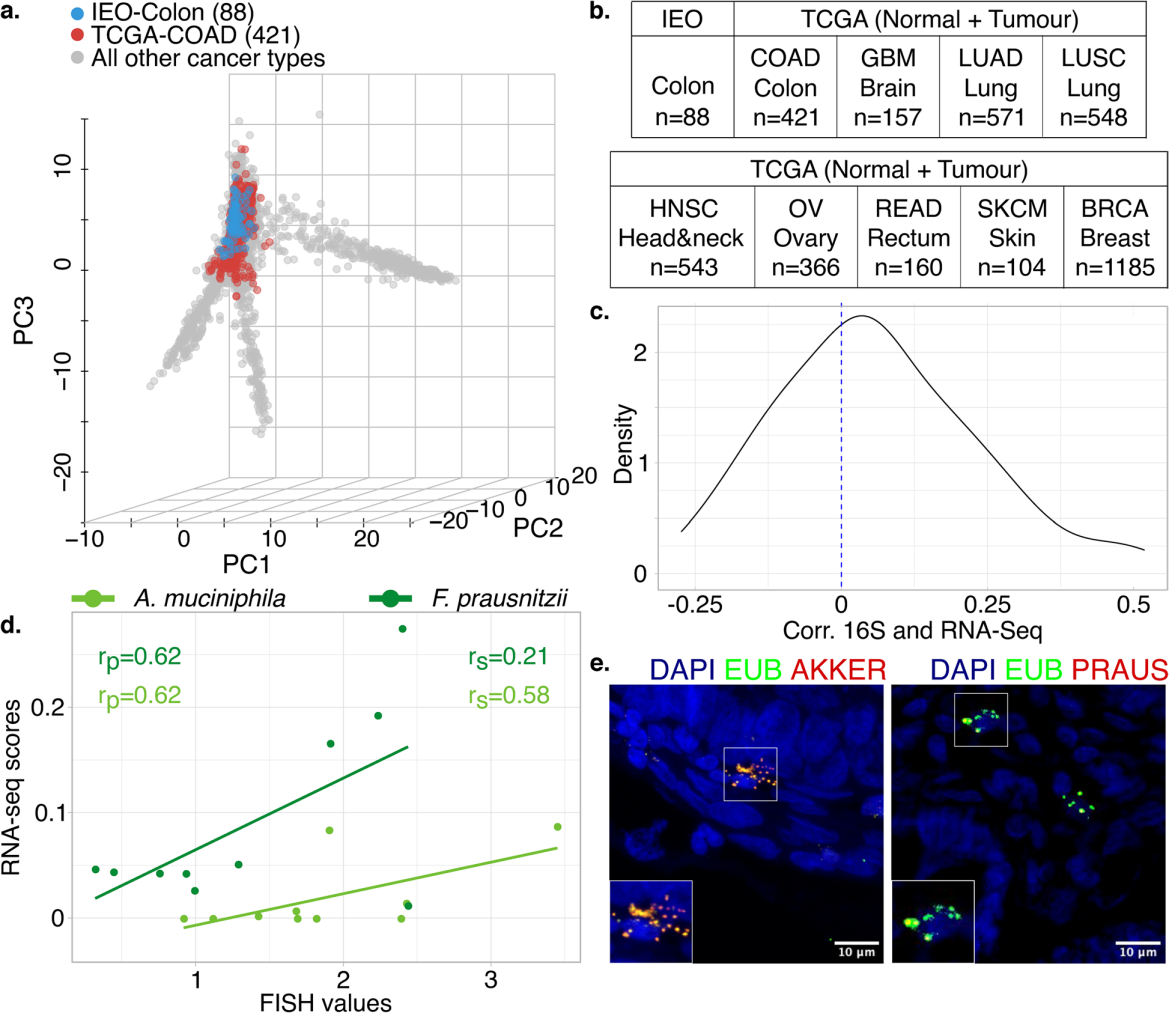
Validation of the reconstructed microbiome profiles from The Cancer Genome Atlas (TCGA) with the European Institute of Oncology (IEO) cohort. **a** Principal component analysis of microbiome profiles from the TCGA (primary tumour and non-pathological solid tissue normal samples) and the IEO cohort shows cross-cohort clustering of colon adenocarcinoma samples. **b** Total number of normal and tumour samples. **c** Density plot of the Spearman coefficients describing the correlation between the 76 most abundant bacterial genera (present in more than 20% of the samples) profiled in both RNA sequencing (RNA-Seq) and ribosomal RNA 16S gene (16S) sequencing data. The majority of correlation coefficients are significantly larger than zero (*p* < 0.005, one-sample Wilcoxon test), meaning a similar tendency of detecting bacteria by the two approaches. **d** Correlation between the reconstructed microbiome (RNA-Seq bacterial relative abundances) and fluorescence in situ hybridisation (FISH) quantification of two bacteria, *Faecalibacterium prausnitzii* and *Akkermansia muciniphila*. Pearson (*r*_*p*_) and Spearman (*r*_*s*_) coefficients are indicated. **e** Representative FISH images of *A. muciniphila* and *F. prausnitzii*. GBM, glioblastoma multiforme; LUAD, lung adenocarcinoma; LUSC, lung squamous cell carcinoma; HNSC, head and neck squamous cell carcinoma; OV, ovarian serous cystadenocarcinoma; READ, rectum adenocarcinoma; SKCM, skin cutaneous melanoma, BRCA, breast invasive carcinoma. Number of samples analysed in brackets

To better understand how accurately our computational approach is able to reconstruct true tissue microbiome composition, we applied the same workflow to a cohort of 30 non-metastatic colon cancer patients who underwent surgical resection at the IEO (Milan). From each patient, we analysed both the tumour and the non-malignant tissues (Additional file 3: Table S3). We first verified that the IEO cohort grouped with TCGA colon samples in microbiome space (Fig. 2a,b). Second, we evaluated the agreement of microbial genus profiles inferred from RNA-Seq data with those generated by sequencing the DNA of bacterial ribosomal RNA 16S from the same samples, an established approach for profiling tissue-resident microbiota. Spearman rank correlation showed good agreement between the two methods when filtering for bacterial genera with a prevalence higher than 20% across samples (Fig. 2c; *p* = 0.004; one-sample Wilcoxon test, *r*_*s*_ = 0.17; Spearman correlation) and the correlation is stronger when considering highly prevalent bacteria (Additional file 1: Fig. S1c). As expected, colon adenocarcinoma samples from TCGA and IEO are grouped together even at the genus-level PCA, see Additional file 1: Fig. S1d. Third, we performed FISH on intestinal tissues from a subset of ten colon cancer patients of the IEO cohort using probes targeting specifically *A. muciniphila* and *F. prausnitzii*, two bacteria found under physiological conditions in the digestive tract [64], see Additional file 1: Fig. S1a-b.

When compared with reconstructed signals obtained by our workflow, the FISH signals showed a good correlation for *A. muciniphila* (*r*_*s*_ = 0.58; Spearman correlation; with a prevalence of 0.5 in RNA-Seq data) and *F. prausnitzii* (*r*_*s*_ = 0.21; Spearman correlation; with a prevalence of 1 in RNA-Seq data) (Fig. 2d,e). Those correlations were not statistically significant. In summary, these analyses indicate that RNA-Seq experiments from human samples can be used to accurately quantify bacterial species, especially those with higher prevalence.

### Association between bacterial composition and clinical and molecular properties of colon cancer

We next wondered if we could use the extracted bacterial reads to detect associations between clinical properties of the tumours and the composition of the microbiome. Considering that the accuracy of the bacterial extraction from RNA-Seq data differs across species, we first tested for associations between coordinates of TCGA samples in the PCA of the microbial abundance space with specific tumour properties. We considered the first six PCs, which together covered more than 10% of the variation in microbiome composition (see “Methods”).

**Fig. 3:**
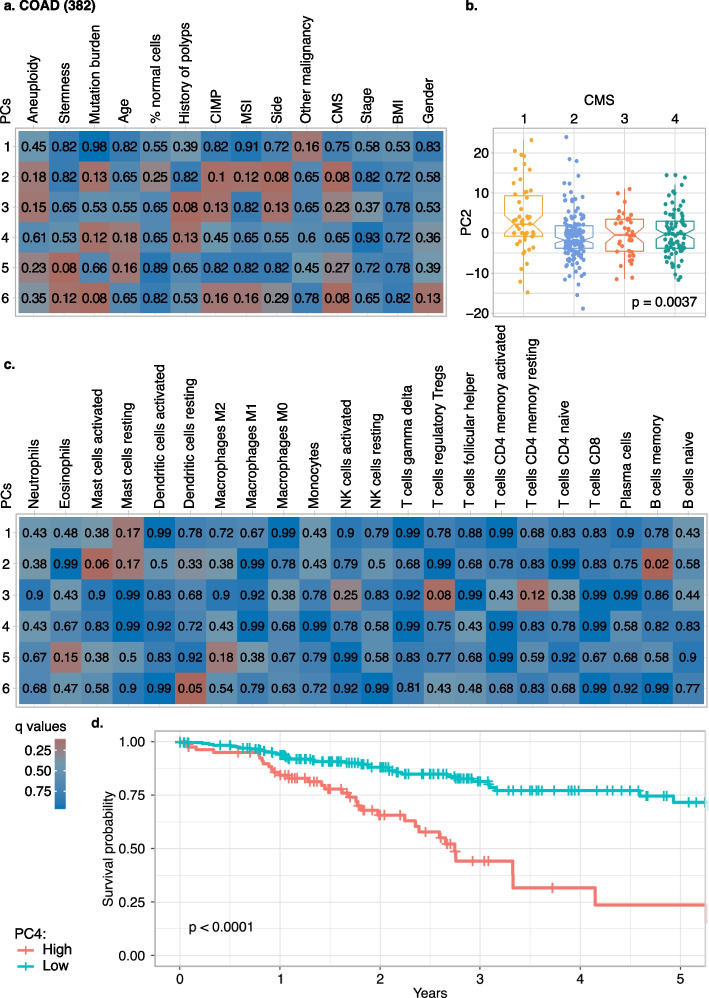
Associations of microbiome profiles reconstructed from colon adenocarcinoma (COAD) with tumour properties. **a** Heatmaps of the *q* values of the association and correlation between the first six principal components (PCs) of COAD microbiome profiles (PCs in rows, clinical properties in columns). **b** Boxplot of PC2 coordinates by consensus molecular subtypes (CMS), highlighting the particular behaviour of CMS1 microbiome profiles. **c** Heatmap analogous to **a** that links the first six microbiome PCs of COAD samples and immune cell infiltration (relative quantification by CIBERSORTx). **d** Kaplan–Meier analysis of disease-free survival on patients stratified by high or low values of PC4. CIMP, CpG methylation phenotype; MSI, microsatellite instability; BMI, body mass index; NK, natural killer. Number of samples analysed in brackets

We initially tested for associations with 14 clinical properties on each analysed cancer type, including age, gender, tumour location (i.e. side), BMI, presence of previous malignancy, history of polyps, stemness of the sample, percentage of normal cells, stage of the tumour, MSI status, CIMP status, CMS (the gene expression–based classification of colon cancer subtypes) [47], aneuploidy status and mutation burden, when available (see “Methods”). We were able to detect associations between the microbiome, as quantified by the microbial PCs, and the properties of the COAD samples (Fig. 3a). For most cancer types, no significant or mildly significant associations (*q* < 0.1 or *q* < 0.2; Wilcoxon, Kruskal–Wallis or Spearman correlation test) were detected, apart from a mild association of mutation burden and stemness in BRCA (*q* = 0.19, Spearman correlation test), see Additional file 1: Fig. S5a-h. Only for COAD the PC coordinates of the reconstructed microbiome showed association with side, CMS, mutation burden, stemness and history of polyps; we also detected mild associations (0.1 < *q* < 0.2 Wilcoxon, Kruskal–Wallis or Spearman correlation test) with MSI status, CIMP status, age, aneuploidy status and gender and other malignancies (Fig. 3a,b and Additional file 1: Fig. S6a-v). To test the robustness of our approach and exclude that our observations could be influenced by technical biases, we repeated the analysis of associations with clinical properties on a small, high-confidence set of species by stringent filtering of the bacteria detected in COAD samples by prevalence and cancer type specificity as well as removing species that co-vary with technical properties of the samples (see “Methods”). We could reconfirm side, MSI and aneuploidy associations with microbial composition based only on 44 species (Additional file 4: Table S4) that could be quantified with high confidence (Additional file 1: Fig. S7a).

Moreover, to reconfirm the associations between the bacterial composition and tumour properties and explore a potential future use of bacterial biomarkers, we wanted to test if the bacterial composition of COAD samples can classify clinical properties. We trained a LASSO logistic regression model on the reconstructed bacterial composition of the COAD samples to classify the tumour properties for which we had sufficient separation in PCA space and a minimum number of samples in each class: side, MSI and CMS (see “Methods”). We predicted MSI and CMS1 samples with an area under the receiver operating characteristic curve (AUC) of 0.7 (Additional file 1: Fig. S8a, c, d) and we classified tumour location (left versus right side of the colon) with a slightly lower AUC of 0.64 (Additional file 1: Fig. S8a, e).

Each colon cancer CMS is characterised by specific molecular and clinical properties and we wondered if this was the case also for their microbial composition. Indeed, microbiome composition varied significantly with CMSs, with CMS1 being linked to a distinct microbiome (Fig. 3b). Since CMS1 is characterised by strong immune cell infiltration and activation of immune evasion pathways, we characterised the immune landscape from gene expression data [52]. Among the 22 immune cell types detected, we found an association between the PCs and the estimates of dendritic cells, memory B cells, regulatory T cells (Tregs) and mast cells in COAD samples (*q* < 0.1; Wilcoxon, Kruskal–Wallis or Spearman correlation tests, see “Methods”) and other mild association with eosinophils, macrophages M2 and resting CD4 memory T cells, both in terms of abundance and proportions of immune cells per sample (Fig. 3c and Additional file 1: Fig. S9a). Moreover, when testing the associations of the bacterial composition with the immune cell proportions of the other cancer types, we detected several interesting associations: Tregs were associated with the majority of the cancer types (COAD, LUAD, LUSC, HNSC and BRCA), followed by dendritic cells (COAD, READ, HNSC and BRCA) and resting CD4 memory T cells (COAD, LUAD, HNSC and BRCA), while monocytes and neutrophils are examples of immune cell subtypes associated with a specific cancer type (BRCA and HNSC respectively), see Additional file 1: Fig. S10 for details. This infiltrating immune cell analysis highlights that some immune cell subtypes are more frequently found associated with the bacterial composition of the tumour while others are more cancer type specific. Along with immune-mediated interactions, the bacteria-host crosstalk relies on mutual metabolic exchanges in both physiologic or pathological conditions. To explore bacterial pathway activity, we quantified the microbial metabolic pathways using a tool to profile the abundance of bacterial metabolic pathways from metagenomics or -transcriptomics data [59]. As our approach revealed substantially fewer bacterial reads than direct bacterial sequencing approaches, we decided to group samples and pool their reads. As tumour side showed one of the strongest associations with the reconstructed microbiomes, we quantified the differential signals of bacterial metabolic pathways in the pooled left versus right colon tumours (Additional file 5: Table S5). After filtering out low-abundance pathways (see “Methods”), we chose those with a differential abundance of at least 30% when comparing the left versus the right side and substantiated their differential abundance via bootstrapping subsets of samples (*q* < 0.2; see “Methods”). This revealed stronger signals for pathways of fatty acid biosynthesis in the left side of the colon, in particular in the palmitate to cis-vaccenate synthesis pathway (we found a higher abundance of (5Z)-dodecenoate biosynthesis I, palmitoleate biosynthesis I (from (5Z)-dodec-5-enoate) and cis-vaccenate biosynthesis) and stearate biosynthesis, in concordance with literature [65–69]. The TCA cycle was associated with the left side of colon. It was previously found enriched in colorectal cancer-associated bacteria [70]. We applied the same approach to CMS and mutation burden since they showed the strongest association with the reconstructed microbiomes together with side. We pooled CMS2, 3 and 4 samples and compared their metabolic pathways with the ones of CMS1 samples but no pathways showed significantly different abundance in one of the two groups (Additional file 5: Table S5). When we compared the pathways of high and low mutation burden samples, we detected two subgroups more abundant in high mutation burden samples: one is associated to DNA degradation (inosine 5'-phosphate, purine ribonucleosides, adenosine and guanosine nucleotides degradation), the other is associated to sugar metabolism (starch, D-glucarate and D-galactarate, GDP-mannose, glucose, glucose-1-phosphate and xylose degradation) (Additional file 5: Table S5).

The genetic background of the host is an essential determinant of both tumour growth and progression and has been suggested to also influence the tumour-associated microbial ecosystem [71, 72]. Thus, we investigated the association of the bacterial composition detected in tumour samples with two molecular properties of tumours: the mutation status of frequently mutated genes (i.e. the driver genes mutation status) and the abnormal number of chromosomes (i.e. the aneuploidy status). We did not detect any significant association between the microbiome composition and the driver gene mutation status in colon cancer [50] (Additional file 1: Fig. S11a). In other cancer types, we did not observe any associations between microbial composition and mutation status of their driver genes either (Additional file 6: Table S6). Likewise, no significant association emerged between extracted microbiome composition and chromosomal gain or loss in COAD (Additional file 1: Fig. S11b, Additional file 7: Table S7 and Additional file 8: Table S8), even though we detected a significant association between the bacterial composition and the general quantification of the degree of aneuploidy, which quantifies the overall deviation from a diploid karyotype, see Fig. 3a. However, we detected an association between microbes and specific chromosome aneuploidy status in HNSC (14, 16 and 20 chromosome loss), OV (alteration of chromosome 14) and READ (chromosome 2 deletion) (Additional file 1: Fig. S12a-c, Additional file 7: Table S7 and Additional file 8: Table S8), even though in this case no significant associations have been detected between the bacterial compositions of these cancer types and the general chromosomal number alteration, see Additional file 1: Fig. S5.

Since molecular and immunological characteristics of the tumour are associated with clinical outcome of colon cancer [73], we next sought a link between the microbial composition extracted from RNA-Seq data and clinical prognosis. Therefore, we fitted Cox proportional-hazard models to the top six PC coordinates and performed univariate analyses assessing the impact of each PC coordinate on OS (Additional file 1: Fig. S13a) and DFS (Additional file 1: Fig. S13b). We found that PC4 was significantly associated with DFS: among the top 20 bacteria contributing to PC4, we found *Cutibacterium granulosum, Corynebacterium tuberculostearicum*, *Moraxella osloensis*, *Gemella haemolysans*, *Staphylococcus epidermidis*, *Finegoldia magna*, *Lawsonella clevelandensis* and *Acinetobacter baumannii*. We then stratified patients into “high” and “low” groups according to PC4 coordinates and applied Kaplan–Meier analysis: patients with higher PC4 coordinates had a higher probability of relapsing (Fig. 3d). Importantly, the survival association was independent of the molecular and clinical properties (e.g. age, polyps history and mutation load) associated with PC4 (multivariate Cox model, Additional file 1: Fig. S13c). We applied the same analysis to the other cancer types but only PC4 of COAD resulted in a significant association with DFS (*q* = 0.03, univariate Cox model), see Additional file 9: Table S9. Together, those results suggest a direct link between microbiome composition and the risk of relapse in COAD samples.

To understand if these associations were detectable only in the tumour microenvironment or reflect a more general dysbiosis of the colon, we tested if the associations between our 14 clinical properties and microbiome composition hold in the non-malignant tissues available from TCGA. The reconstructed microbiome of these non-malignant samples of the colon did not show an association with the clinical properties available (Additional file 1: Fig. S14a). However, the lack of significance might be due to lower statistical power (TCGA contains only 39 non-malignant colon samples). To exclude this possibility, we tested the same associations in the subset of tumour samples paired with the non-malignant ones. For seven of the previous ten significant associations, we observed lower significance levels in the paired, reduced tumour cohort compared to non-malignant, showing a tendency of association similar to the one detected with the full COAD cohort (Additional file 1: Fig. S14b). Even though we could not rule out that significant associations may be seen with a larger number of non-malignant samples, the complete absence of associations suggests that the detected ones in our initial pool of samples are tumour-microenvironment specific. Given the absence of associations detected in the non-malignant COAD samples, we wondered if the bacterial composition of samples could be used to distinguish between non-malignant and tumour samples. We trained another LASSO regression model to classify the status (non-malignant versus tumour) of samples based on the reconstructed bacterial composition: using the 39 non-malignant samples and their paired tumour counterparts, we reached an AUC of 0.83 (Additional file 1: Fig. S8b,f), highlighting that we can predict the malignancy status of the sample from its reconstructed bacterial composition.

### Identification of bacteria associated with specific cancer-related properties

**Fig. 4:**
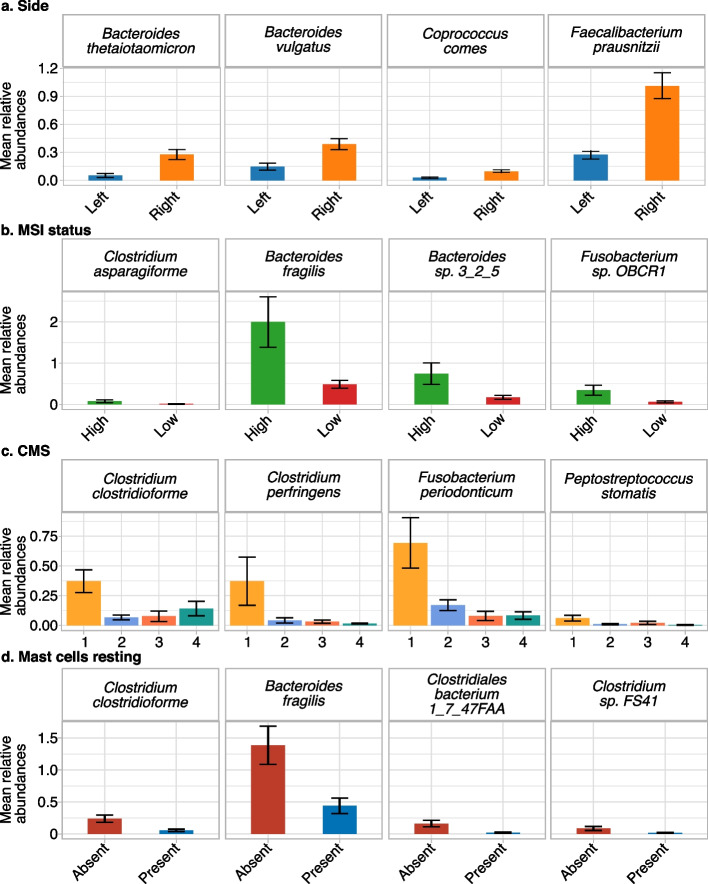
Species associated with colon adenocarcinomas properties. Barplots of the means of the bacterial relative abundances of a subset of the bacterial species with differential distribution in **a** the side, **b** microsatellite instability (MSI) level, **c** consensus molecular subtype (CMS) and **d** mast cells resting. In total, we found nine bacteria associated with side, five with MSI, eighteen with CMSs and twelve with resting mast cells. Error bars showing the standard error

Our previous analyses revealed links between microbiome composition and different properties of colon tumours. To further refine this, we investigated if specific bacteria were associated with each of the different properties. To identify the species associated with the left- or right-sided tumours, we tested for abundance differences for a subset of colon-specific species (cancer type–specific and prevalent in colon cancer samples, see “Methods”), while controlling for the technical variation (independence test blocking by plate ID; see “Methods”) (Fig. 4a). We found nine species whose abundances differed between the left and the right side of the colon. In particular, we discovered that *F. prausnitzii*, *Coprococcus comes* and two *Bacteroides* spp. (*Bacteroides vulgatus* and *Bacteroides thetaiotaomicron*) showed higher abundances in the samples from the right (Fig. 4a and Additional file 10: Table S10). Notably, these four bacteria were among the 20% of species contributing the most to PC2, the most robust side-associated PC (Fig. 3a). In addition, in the previously described LASSO regression model, *F. prausnitzii* showed a high relative weight on the right side of the colon (Additional file 1: Fig. S8e). When the same approach was applied to the other properties significantly associated with PCs (*q* < 0.2; Wilcoxon, Kruskal–Wallis or Spearman correlation tests), CMS and MSI status were the only two showing an association with specific bacterial taxa. Specifically, five species had a higher abundance in MSI high samples (Additional file 10: Table S10), including *Bacteroides fragilis*, *Clostridium asparagiforme*, *Fusobacterium sp. OBRC1* and *Bacteroides sp. 3_2_5* (Fig. 4b), which were strongly contributing to the two PCs associated with MSI level (PC2 and PC6). *B. fragilis*, *C. asparagiforme* and *F. sp. OBRC1* were also highlighted by the LASSO regression model as a marker of MSI high samples (Additional file 1: Fig. S8c). We also tested which bacteria were associated with the highly immune infiltrated CMS1 subtype (Fig. 4c and Additional file 10: Table S10) and found 18 bacteria from *Clostridium*, *Bacteroides*, *Fusobacterium, Actinomyces* and *Peptostreptococcus* genera, and *Firmicutes* phylum. Even if not *F. nucleatum* itself, which has been previously linked to the growth and progression of colorectal cancer [73–75], we detected five *Fusobacterium* species with a higher level in the CMS1 subgroup. These bacteria contributed to PC2 or PC6, the two PCs associated with CMS. Moreover, five species of *Clostridium* were found associated with CMS1: while *Clostridium perfringens* was not contributing to PC2, the LASSO model used it to classify CMS1 samples, together with *Fusobacterium periodonticum* and *F. sp. OBRC1* (Additional file 1: Fig. S8d).

A similar approach was applied to detect potential associations of specific bacteria to subtypes of immune cells. In this regard, 12 bacteria were associated with the absence of resting mast cells (with an opposite tendency for activated mast cells): *B. fragilis*, *Clostridium clostridioforme*, *Clostridiales bacterium 1_7_47FAA* and *Clostridium sp. FS41* (Fig. 4d and Additional file 10: Table S10). All of them contributed to PC2, which was associated with mast cell infiltration, and *C. clostridioforme* was among the bacteria associated with CMS1.

Finally, we tested if there were any specific bacteria associated with patient survival. Given the association of PC4 with the DFS of COAD patients, we selected 100 bacteria with the highest PC4 loading values and performed a univariate Cox analysis. Seventeen bacteria showed significant positive associations with the relapse probability (hazard ratio > 3; *q* < 0.2; Wald test; Additional file 10: Table S10), including *Corynebacterium matruchotii*, *A. baumannii*, *Pseudomonas stutzeri* and *Propionibacterium namnetense*.

Even if we did not identify strong associations between the bacterial composition and tumour properties of the other cancer types, we anyway tested if any single bacteria were associated with clinical properties. We selected the most prevalent bacteria (present in 10% or more samples, see “Methods”) and tested their association to tumour properties in all the investigated cancer types (Additional file 1: Fig. S15). LUAD, HNSC, OV and BRCA showed at maximum two properties with associated species, READ four, compared to COAD with six properties being associated with differential species abundance, see Additional file 11: Table S11.

Given the associations of single bacterial species with tumour properties in COAD, we wondered if we could identify associations even at the genus level. On the genus level, we assessed the reliability of quantification by comparing 16S and RNA-Seq data. As shown in the Additional file 1: Fig. S16, we detected 18 high-confidence genera (Spearman R > 0.25, Additional file 1: Fig. S16) and tested their association with clinical properties. We detected seven associated properties and all of them were previously observed at the species level. With the PCA approach at the species level, we detected a higher number of significant associations (*q* < 0.2; 22 significant *q*) than with a single genera approach (16 significant *q*). Some associations such as with MSI and CIMP were missing at the genus level, hinting at a lower sensitivity when using genera and suggesting that these could be species-specific associations.

## Discussion

Tumour development mirrors species evolution in the sense that tumours acquire random alterations that may confer a fitness advantage or disadvantage to the clones that carry the alteration. While in species evolution the modulatory effect of the environment on fitness and hence selection is well established (e.g. the shape and size of the beak of Darwin finches is an adaption to availability of food), the link between the ecological niche in which tumours grow and selective forces favouring specific phenotypes is much less clear. Our present work focuses on detecting different microbes in the tumour niche, intending to identify how differences in bacterial composition are associated with specific tumour properties.

Heterogeneity in both the human microbiome composition and tumour molecular properties poses a challenge for association studies of bacteria and tumour properties as it requires a large number of samples. Our approach allowed us to overcome this problem since we were able to deeply portray the clinical and molecular properties of every tumour, taking advantage of the high amount of information and analyses of TCGA cohort patients and, concurrently, reconstruct the bacterial composition of each sample analysed. Moreover, the possibility of observing and correcting the technical differences of the reconstructed microbiomes prevents the detection of spurious associations. This batch correction workflow makes it possible in principle to combine these analyses with other datasets to further increase statistical power by leveraging the large amount of human RNA-Seq experiments that are publicly available.

The composition of bacteria that we reconstructed from human RNA-Seq data is affected by biases and contamination thus limiting our capability to investigate the bacterial ecosystem of the tumour. The impact of some of these biases can be quantified from the association of the bacterial composition with technical features that are reported by TCGA. Our computational approach detects and corrects for the most strongly associated feature. Technical features are highly correlated among each other and, thereby, when correcting for the strongest associated feature, our approach substantially reduces the impact of other technical features too. Still, we cannot rule out that weaker or undetected batch effects remain and may still influence our observations. In addition, it is sometimes difficult to decide if a reported property of the tumour is in fact a technical or biologically relevant feature (e.g. the percentage of normal cells).

Indeed, further validations and improvements may be needed for accurately profiling intratumoural bacteria as our approach cannot perfectly mirror the actual abundance of each single bacterium as it is the case with metagenomics methods that show a large variability in accuracy across methods [76]. Using PCA allowed us to robustly capture prominent trends of variation in bacterial composition and circumvents the need to quantify every single bacterial taxon accurately. With this integrated workflow and the systematic analysis of a total of 264 tumour properties, we went beyond previous work which established links involving particular tumour properties, species or tumour types [19–28]. This confirmed previous observations (e.g. CMS, clinical outcome) and revealed novel associations (e.g. aneuploidy status).

Besides these global relationships with bacterial composition, we detected specific bacteria associated with those tumour properties. Some of them have already been associated with colon cancer and inflammation or gastrointestinal diseases [47, 77–83] while others have been identified in healthy colon [84, 85]. In particular we identified *B. fragilis*, some strains of which are known commensals of the human gut, whereas others can enhance tumour growth via production of an enterotoxin that commensal strains are lacking. This enterotoxin can induce tumours in several ways, one of them includes immune cell deregulation [86]. While among the healthy related bacteria, we found *F. prausnitzii* enriched in the right side of the colon: *F. prausnitzii* is also known as one of the main anaerobic bacteria that feed the colon cells by fermentation [84], which is one of the main roles of microbiota in the proximal colon [87]. The differences in the sides of the colon were also evident in the modification of the chemical context of the tumour [65]: for example, we showed the differences in bacterial fatty acid metabolism of the tumours from the two sides of the colon. Interestingly, some of the pathways associated with the left part of the colon were previously associated with cancer [65–68] or inflammation [69]. In particular, palmitate accumulation has been shown to contribute to creating an immune-suppressive tumour microenvironment [88]. In this context, the immune system involvement represents another mechanism connecting bacteria with tumour properties: previous studies have demonstrated that the interaction between bacteria and the immune system can shape the growth and progression of specific tumour subtypes [11]. Here, mast cells were most strongly linked with bacteria amidst all tested immune cell subtypes. With the advantage of using human colon cancer samples, this result confirms previous studies demonstrating that bacteria can induce mast cell activation in mouse models or in small cohorts of patients [89, 90]. Moreover, the infiltrating immune cells are associated with the tumoural bacterial composition not only in colon cancer, but also in other cancer types. Interestingly, for these tissues few other bacterial composition associations were detected. We observe that some immune cell subtypes are more frequently associated to the bacterial composition of the tumour, e.g. the Tregs and dendritic cells. These two cells are known to be reactive to microbial stimuli and play a role in microbial regulation [91, 92]. Moreover, we identified different types of DNA degradation pathways associated with mutation burden of the tumour (e.g. purine degradation). This highlights an interesting association between bacterial metabolism and mutational processes that should be further explored.

These differences can directly affect (or be affected by) the properties of the tumour since they shape its chemical environment: interactions through immune cells and metabolism can be used to describe and represent the tumour niche as an evolving ecosystem.

Despite clinical prognosis in colorectal cancer depending strongly on the time of diagnosis, almost half of the resected colon tumours relapse within 5 years from surgery [93]. Here we showed that the bacterial composition of tumours can be predictive of patient prognosis. Among the bacteria we identified, *A. baumannii*, *P. namnetense* and *P. stutzeri* associated with bad prognosis and have been previously linked to human diseases or cancer [94–97].

In our analysis, we decided to focus on the association between colon cancer properties and microbiome composition. While we observed particularly strong associations in colon, we would like to clarify that we also observed weaker associations in other cancer types (e.g. breast). Those should be explored further in future studies. While other studies observed the specific association between colon cancer properties such as survival and microbiome composition too [19, 20, 26], we here systematically test a large number of molecular and clinical features and, therefore, expand beyond the previous work. The large number of detected associations might reflect the specific quantity and diversity of the colon microbiome and its direct influence on the colon [98, 99].

Despite the clear clinical relevance of some of our observations (such as associations of the bacterial composition of tumour to patient’s prognosis), the primary aim of this study is to better understand the tumour and its environment as a system where the probability of occurrence of components are statistically linked to each other. Further experimental work would be needed to address directionality and causality of the described associations. However, our work indicates that the microbial component of the tissue microenvironment might influence selection in tumour evolution and outcome.

## Conclusions

By showing that specific consortia of bacteria are associated explicitly with molecular and clinical properties of the tumour, we suggest that the profiling of bacterial composition can be developed into a stratification biomarker, with relevant implications in prognosis predictions and with the potential to implement colon cancer therapies, e.g. immune checkpoint inhibitor therapy.

## Supplementary Information

**Additional file 1: Table S1 and supplementary figures.** FISH primers, 16S probes and all supplementary figures.

**Additional file 2: Table S2.** COAD PC loadings. Bacterial species involved in the first six COAD PCs.

**Additional file 3: Table S3.** IEO cohort clinical metadata summary.

**Additional file 4: Table S4.** COAD specific bacterial species. Highly prevalent and cancer-type specific species of COAD (not affected by technical bias).

**Additional file 5: Table S5.** HUMAnN3 microbial pathways.

**Additional file 6: Table S6.** Mutated gene associations. Association tests (q values) between each of the first six PCs from the COAD reconstructed microbiome and the gene mutational status.

**Additional file 7: Table S7.** Whole chromosome associations. Association tests (q values) between each of the first six PCs from the COAD reconstructed microbiome and the whole chromosome aneuploidy status.

**Additional file 8: Table S8.** Chromosome arm level associations. Association tests (q values) between each of the first six PCs from the COAD reconstructed microbiome and the arm level chromosome aneuploidy status.

**Additional file 9: Table S9.** Survival analyses. DFS and OS univariate Cox survival analysis results of all the cancer types analysed.

**Additional file 10: Table S10.** Species associated with COAD properties. Filtered bacteria tested for differential abundances in the sublevels of the relevant tumour COAD properties.

**Additional file 11: Table S11.** Species associations for other cancer types. Most prevalent bacteria tested for differential abundances in the sublevels of the cancer type properties.

## Data Availability

The raw, controlled access data from the IEO cohort are available on request from the European Genome-Phenome Archive (EGA) EGAD00001009635, (https://ega-archive.org/datasets/EGAD00001009635) [100]. TCGA raw data are available from NCI Genomic Data Commons (GDC). The microbiome data generated and analysed in this study, any additional methods, source data and codes to reproduce these results are available at https://github.com/SamGa3/microbiome_reconstruction [29].

## Abbreviations

WXS: Whole exome sequencing
RNA-Seq: RNA sequencing
TCGA: The Cancer Genome Atlas
GDC: NCI Genomic Data Commons
COAD: Colon adenocarcinoma
GBM: Glioblastoma multiforme
LUAD: Lung adenocarcinoma
LUSC: Lung squamous cell carcinoma
HNSC: Head and neck squamous cell carcinoma
OV: Ovarian serous cystadenocarcinoma
READ: Rectum adenocarcinoma
SKCM: Skin cutaneous melanoma
BRCA: Breast invasive carcinoma
IEO: European Institute of Oncology
16S: Ribosomal RNA 16S gene
FISH: Fluorescence in situ hybridisation
DAPI: 4’,6-Diamidino-2-phenylindole
PBS: Phosphate buffered saline
BMI: Body mass index
MSI: Microsatellite instability
CIMP: CpG methylation phenotype
CMS: Consensus molecular subtype
TPM: Transcript per million
FPKM: Fragments per kilobase million
PCs: Principal components
PCA: Principal component analysis
plate ID: 96-Well plate identifier
LASSO: Least absolute shrinkage and selection operator
SD: Standard deviation
CPM: Copies per million
DFS: Disease-free survival
OS: Overall survival
FDR: False discovery rate
AUC: Area under the receiver operating characteristic curve
Tregs: Regulatory T cells
NK: Natural killer cells
