# Supplementary material for: Location and condition based reconstruction of colon cancer microbiome from human RNA sequencing data
Source: Genome Med. 2023 May 2;15:32. doi: 10.1186/s13073-023-01180-9 (PMC10155404; doi:10.1186/s13073-023-01180-9)
Supplement: Supplementary file 1 — Additional file 1: Table S1 and supplementary figures. FISH primers, 16S probes and all supplementary figures. [file 13073_2023_1180_MOESM1_ESM.pdf]

Sambruni et al.

Location and condition based reconstruction of colon cancer microbiome from human RNA sequencing data

FISH probes:

GCTGCCTCCCGTAGGAGT	EUB338	CY3	Eubacteria
CCTTGCGGTTGGCTTCAGAT	MUC1437	CY5	*Akkermansia muciniphila*
GTGCCCAGTAGGCCGCCTTC	FP698	CY5	*Faecalibacterium prausnitzii*

16S Primers:

forward	5'-TCGTCGGCAGCGTCAGATGTGTATAAGAGACAGCCTACGGGNGGCWGCAG-3'
reverse	5'-GTCTCGTGGGCTCGGAGATGTGTATAAGAGACAGGACTACHVGGGTATCTAATCC-3'

Table S1. Tables with the sequences of the Fluorescence in situ hybridisation (FISH) probes and the primers for ribosomal RNA 16S gene (16S) sequencing.

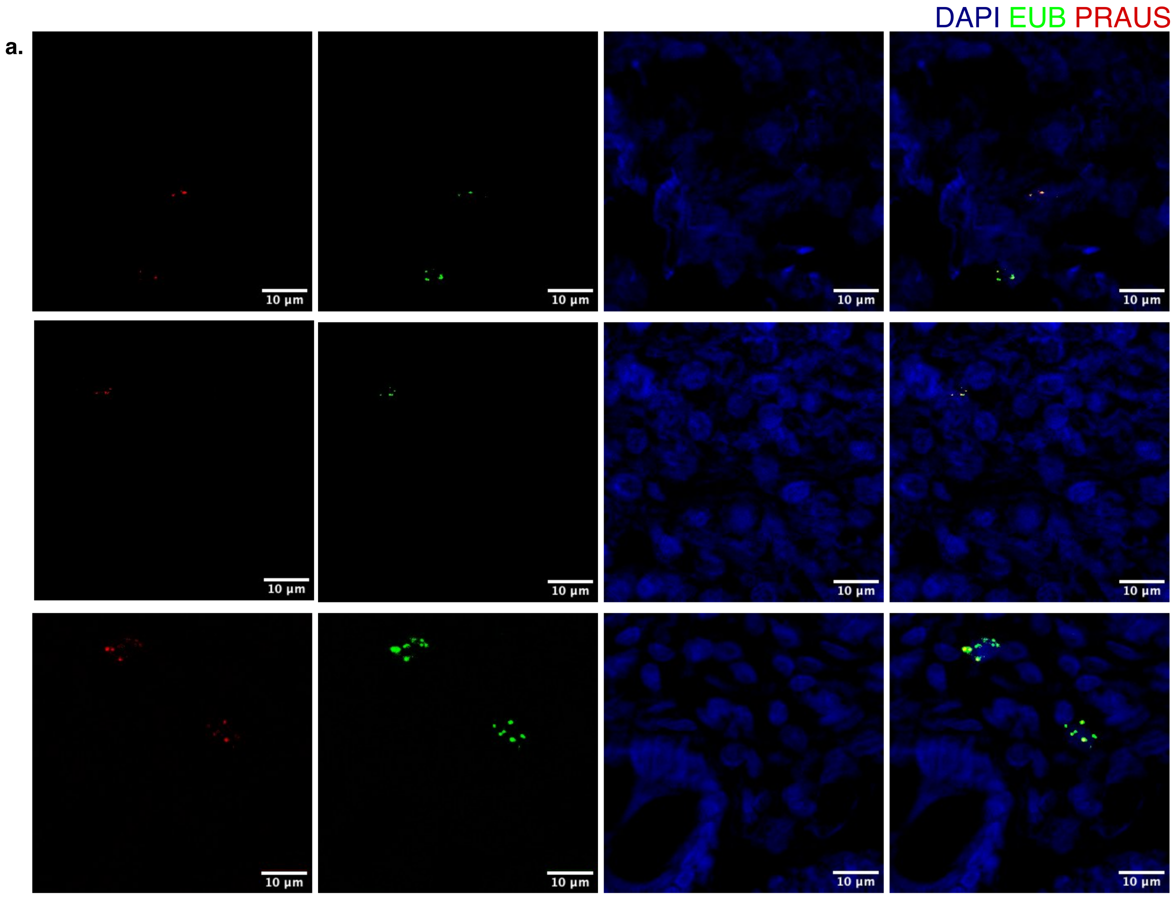
DAPI EUB PRAUS
a.
10 µm

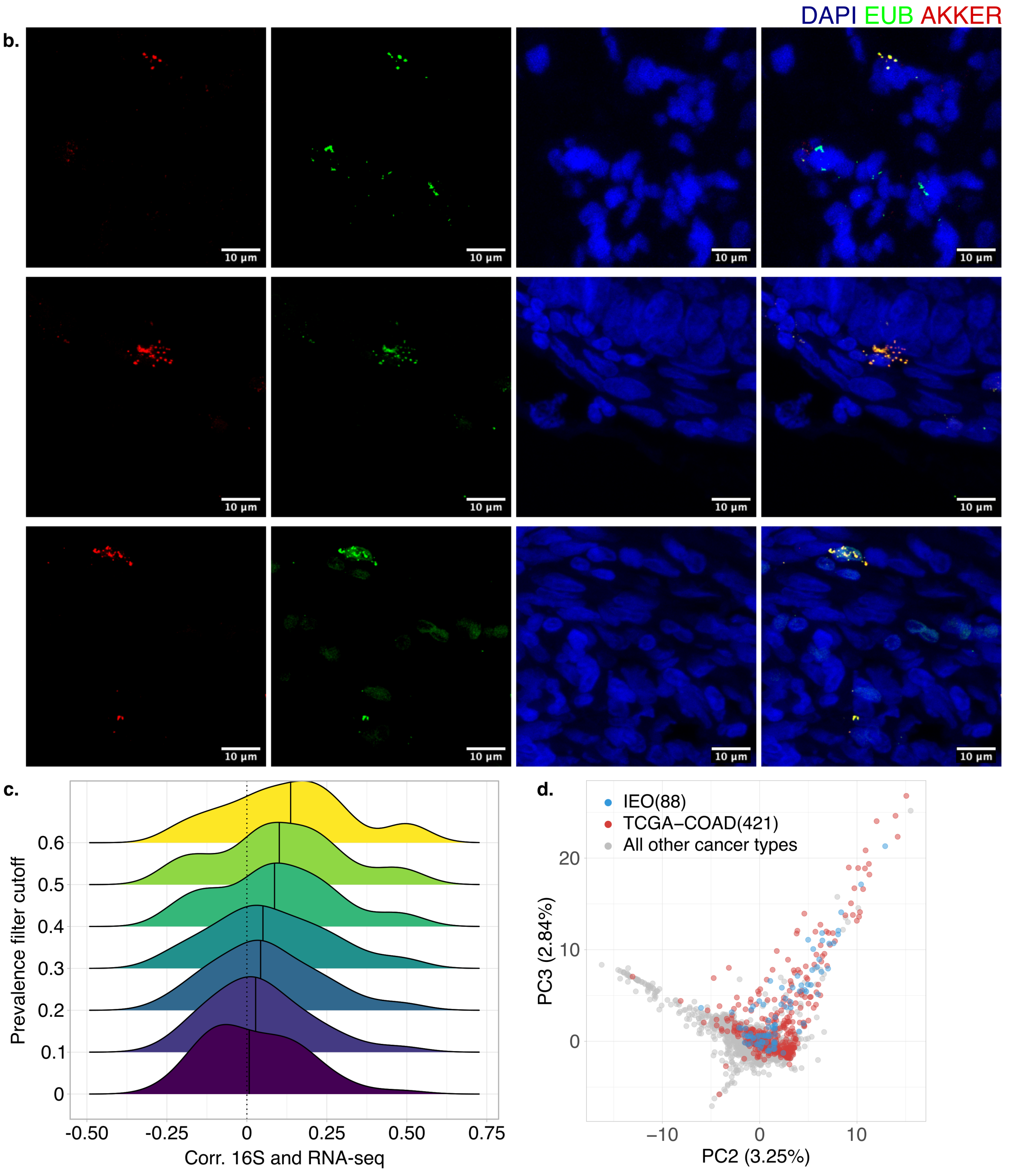

Fig S1. Fluorescence in situ hybridisation images of bacteria (EUB, green), 4',6-Diamidino-2-phenylindole (DAPI, blue) and (a) *Faecalibacterium prausnitzii* or (b) *Akkermansia muciniphila* (red). The right column shows the merge of the 3 pictures on the left. (c) Ridge plot showing the distributions of the Spearman coefficients of the correlations of the bacterial genera quantifications by RNA sequencing (RNA-Seq) and ribosomal RNA 16S gene (16S) sequencing, at different filter cutoffs for

bacterial presence. From the thresholds of 0 to 60% prevalence of bacteria detected by both the methods, there is a general increase of coefficient values with a distribution significantly over zero ($p < 0.05$; one-sample Wilcoxon test). (d) Principal component analysis of the most variable bacterial genera (200 genera) microbiome profiles from the The Cancer Genome Atlas (TCGA) (primary tumour and non-pathological solid tissue normal samples) and the European Institute of Oncology (IEO) cohort shows cross-cohort clustering of colon adenocarcinoma samples. COAD, colon adenocarcinoma.

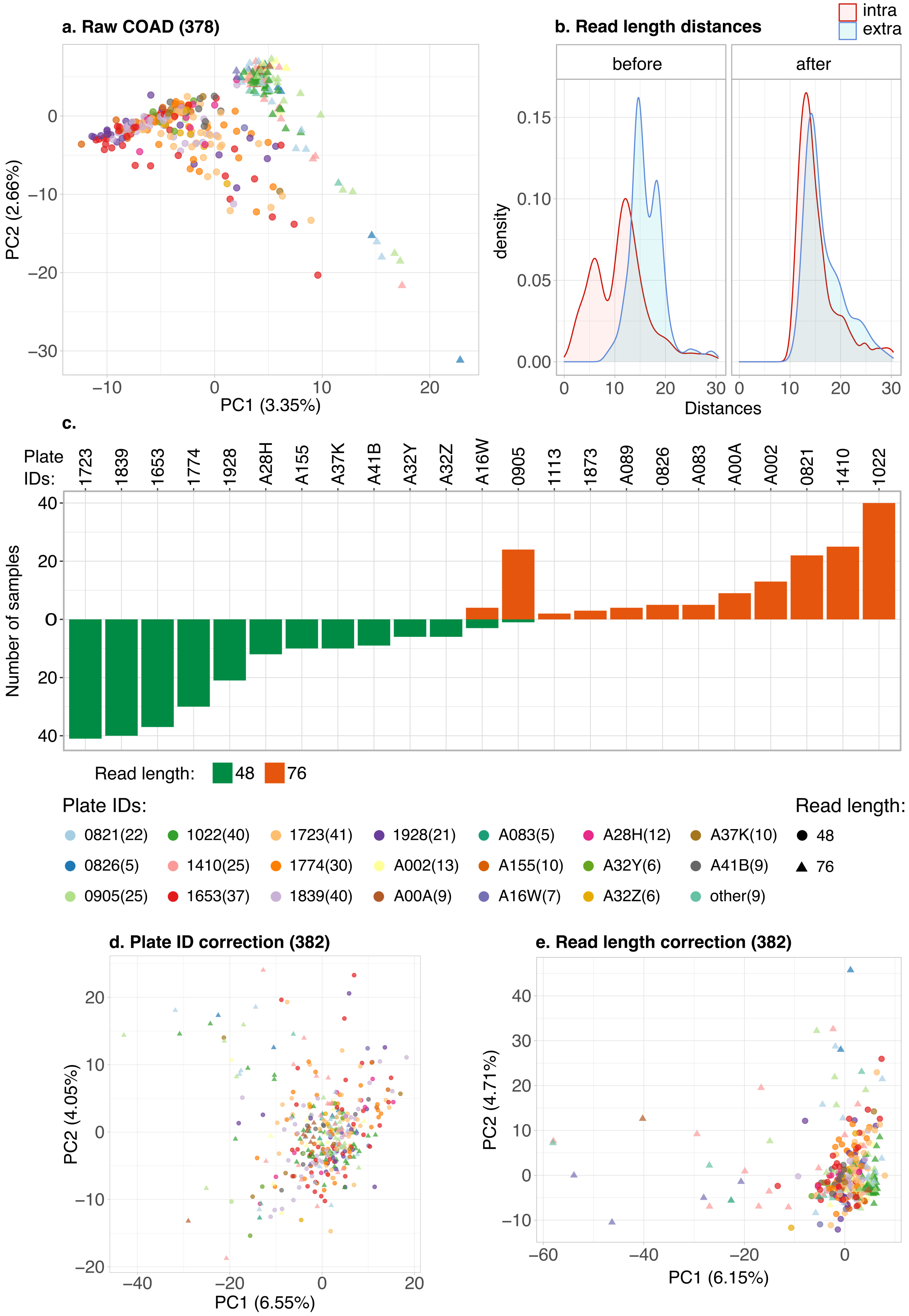

a. Raw COAD (378)
PC2 (2.66%)
PC1 (3.35%)
b. Read length distances
intra
extra
before
after
density
Distances
c.
Plate IDs:
1723
1839
1653
1774
1928
A28H
A155
A37K
A41B
A32Y
A32Z
A16W
0905
1113
1873
A089
0826
A083
A00A
A002
0821
1410
1022
Number of samples
Read length:
48
76
Plate IDs:
0821(22)
1022(40)
1723(41)
1928(21)
A083(5)
A28H(12)
A37K(10)
0826(5)
1410(25)
1774(30)
A002(13)
A155(10)
A32Y(6)
A41B(9)
0905(25)
1653(37)
1839(40)
A00A(9)
A16W(7)
A32Z(6)
other(9)
Read length:
48
76
d. Plate ID correction (382)
PC2 (4.05%)
PC1 (6.55%)
e. Read length correction (382)
PC2 (4.71%)
PC1 (6.15%)

Fig S2. (a) Principal component analysis (PCA) on the raw reconstructed microbiome of colon adenocarcinoma (COAD) samples. Each colour corresponds to a different plate, the shape represents the read length used to analyse the samples (48 or 76 bp, dot and triangle respectively). (b) Comparison of the distances between colon adenocarcinoma (COAD) and rectum adenocarcinoma (READ) samples analysed with the same read length (intra, red) and the distances between samples analysed with different read length (extra, blue), shown before and after the batch correction by plate. Even if we corrected by another technical bias (the plate ID), the read length effect bias was reduced. (c) Number of COAD samples per plate ID and read length. PCA on the COAD reconstructed microbiome corrected by (d) plate ID and (e) read length. PC, principal component. Number of samples analysed in brackets.

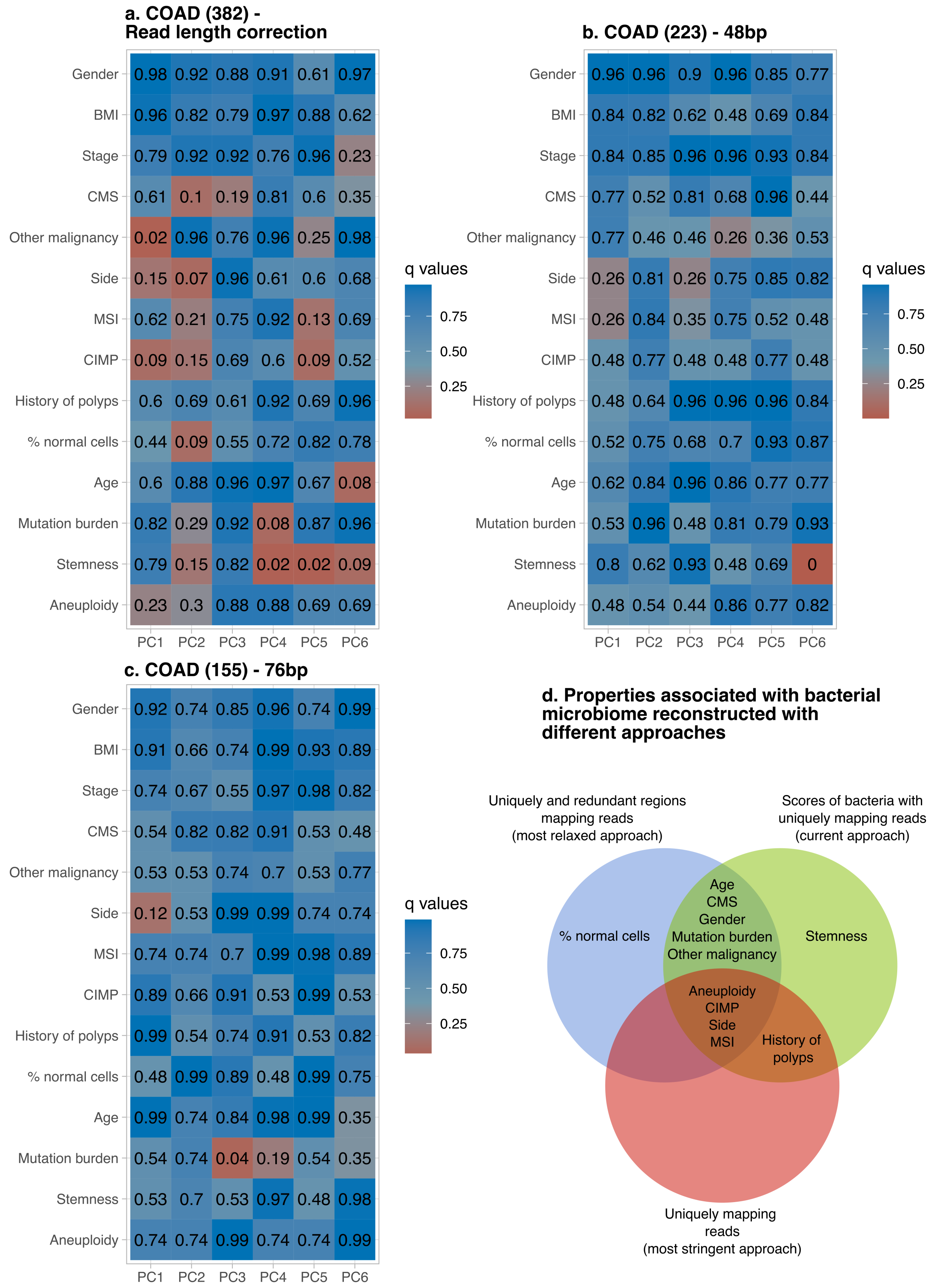
a. COAD (382) - Read length correction
Gender 0.98 0.92 0.88 0.91 0.61 0.97
BMI 0.96 0.82 0.79 0.97 0.88 0.62
Stage 0.79 0.92 0.92 0.76 0.96 0.23
CMS 0.61 0.1 0.19 0.81 0.6 0.35
Other malignancy 0.02 0.96 0.76 0.96 0.25 0.98
Side 0.15 0.07 0.96 0.61 0.6 0.68
MSI 0.62 0.21 0.75 0.92 0.13 0.69
CIMP 0.09 0.15 0.69 0.6 0.09 0.52
History of polyps 0.6 0.69 0.61 0.92 0.69 0.96
% normal cells 0.44 0.09 0.55 0.72 0.82 0.78
Age 0.6 0.88 0.96 0.97 0.67 0.08
Mutation burden 0.82 0.29 0.92 0.08 0.87 0.96
Stemness 0.79 0.15 0.82 0.02 0.02 0.09
Aneuploidy 0.23 0.3 0.88 0.88 0.69 0.69
PC1 PC2 PC3 PC4 PC5 PC6
q values
0.75
0.50
0.25
b. COAD (223) - 48bp
Gender 0.96 0.96 0.9 0.96 0.85 0.77
BMI 0.84 0.82 0.62 0.48 0.69 0.84
Stage 0.84 0.85 0.96 0.96 0.93 0.84
CMS 0.77 0.52 0.81 0.68 0.96 0.44
Other malignancy 0.77 0.46 0.46 0.26 0.36 0.53
Side 0.26 0.81 0.26 0.75 0.85 0.82
MSI 0.26 0.84 0.35 0.75 0.52 0.48
CIMP 0.48 0.77 0.48 0.48 0.77 0.48
History of polyps 0.48 0.64 0.96 0.96 0.96 0.84
% normal cells 0.52 0.75 0.68 0.7 0.93 0.87
Age 0.62 0.84 0.96 0.86 0.77 0.77
Mutation burden 0.53 0.96 0.48 0.81 0.79 0.93
Stemness 0.8 0.62 0.93 0.48 0.69 0
Aneuploidy 0.48 0.54 0.44 0.86 0.77 0.82
PC1 PC2 PC3 PC4 PC5 PC6
q values
0.75
0.50
0.25
c. COAD (155) - 76bp
Gender 0.92 0.74 0.85 0.96 0.74 0.99
BMI 0.91 0.66 0.74 0.99 0.93 0.89
Stage 0.74 0.67 0.55 0.97 0.98 0.82
CMS 0.54 0.82 0.82 0.91 0.53 0.48
Other malignancy 0.53 0.53 0.74 0.7 0.53 0.77
Side 0.12 0.53 0.99 0.99 0.74 0.74
MSI 0.74 0.74 0.7 0.99 0.98 0.89
CIMP 0.89 0.66 0.91 0.53 0.99 0.53
History of polyps 0.99 0.54 0.74 0.91 0.53 0.82
% normal cells 0.48 0.99 0.89 0.48 0.99 0.75
Age 0.99 0.74 0.84 0.98 0.99 0.35
Mutation burden 0.54 0.74 0.04 0.19 0.54 0.35
Stemness 0.53 0.7 0.53 0.97 0.48 0.98
Aneuploidy 0.74 0.74 0.99 0.74 0.74 0.99
PC1 PC2 PC3 PC4 PC5 PC6
q values
0.75
0.50
0.25
d. Properties associated with bacterial microbiome reconstructed with different approaches
Uniquely and redundant regions mapping reads (most relaxed approach)
Scores of bacteria with uniquely mapping reads (current approach)
Uniquely mapping reads (most stringent approach)
% normal cells
Age
CMS
Gender
Mutation burden
Other malignancy
Stemness
Aneuploidy
CIMP
Side
MSI
History of polyps

Fig S3. (a) Heatmaps of the *q* values of the associations and correlation between the first six principal components (PCs) of the principal component analysis (PCA) on the reconstructed microbiome and the clinical properties from the metadata of colon adenocarcinoma (COAD) samples, applying the read length batch correction, showing similar bacterial component association to the one corrected by plate ID. Same analysis with the reconstructed microbiome of only COAD samples sequenced with read length of (b) 48 or (c) 76 bp. (d) Venn diagram of the tumour properties detected as significantly associated with the bacterial composition reconstructed with three different approaches in COAD. CIMP, CpG methylation phenotype; MSI, microsatellite instability; BMI, body mass index. Number of samples analysed in brackets.

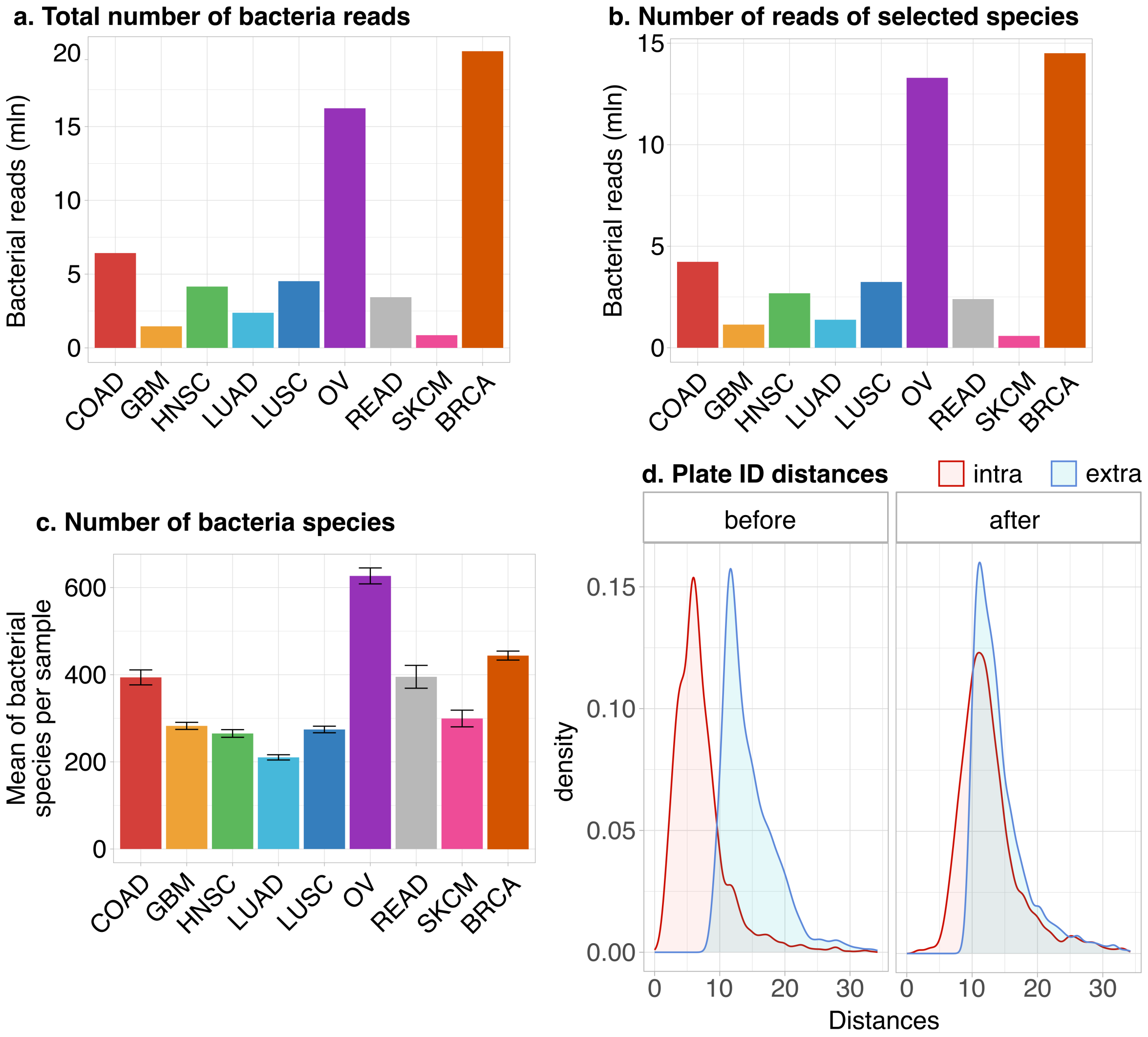

Fig S4. (a) Total bacterial reads detected by Pathseq in the RNA sequencing bam files of the tissues analysed. (b) Number of bacterial reads of the selected species for the principal component analysis in each cancer type. (c) Per-sample means of the bacterial species detected after bacterial relative abundances correction for non-redundant reads. Error bars showing the standard error. (d) Comparison of the distances between colon adenocarcinoma (COAD), glioblastoma multiforme (GBM), lung adenocarcinoma (LUAD), lung squamous cell carcinoma (LUSC), head and neck squamous cell neoplasms (HNSC), ovarian serous cystadenocarcinoma (OV), rectum adenocarcinoma (READ), skin cutaneous melanoma (SKCM) and breast invasive carcinoma (BRCA) samples analysed in the same plate (intra, red) and the distances between samples analysed in different plates (extra, blue), shown before and after the batch correction by plate.

Sambruni et al.

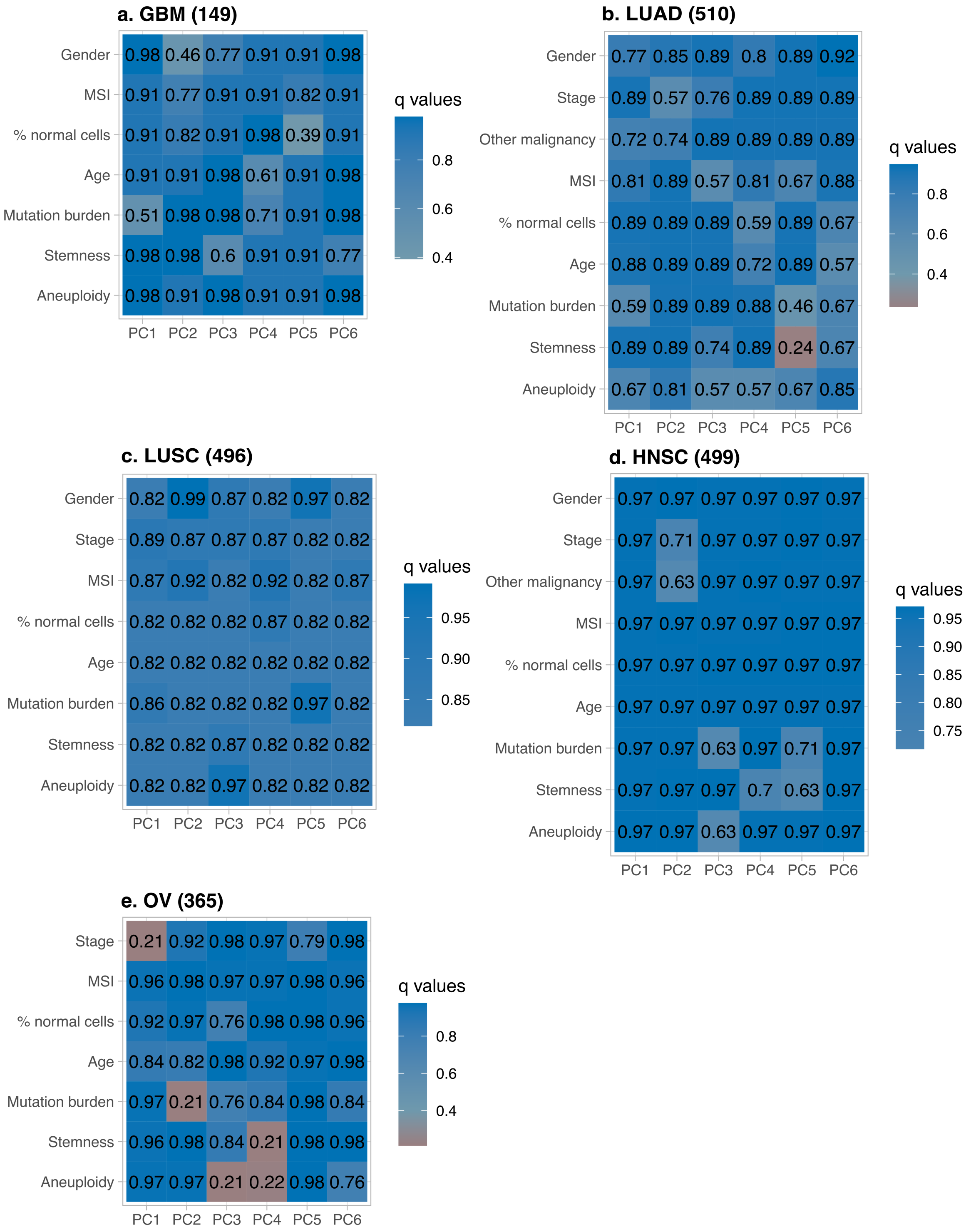

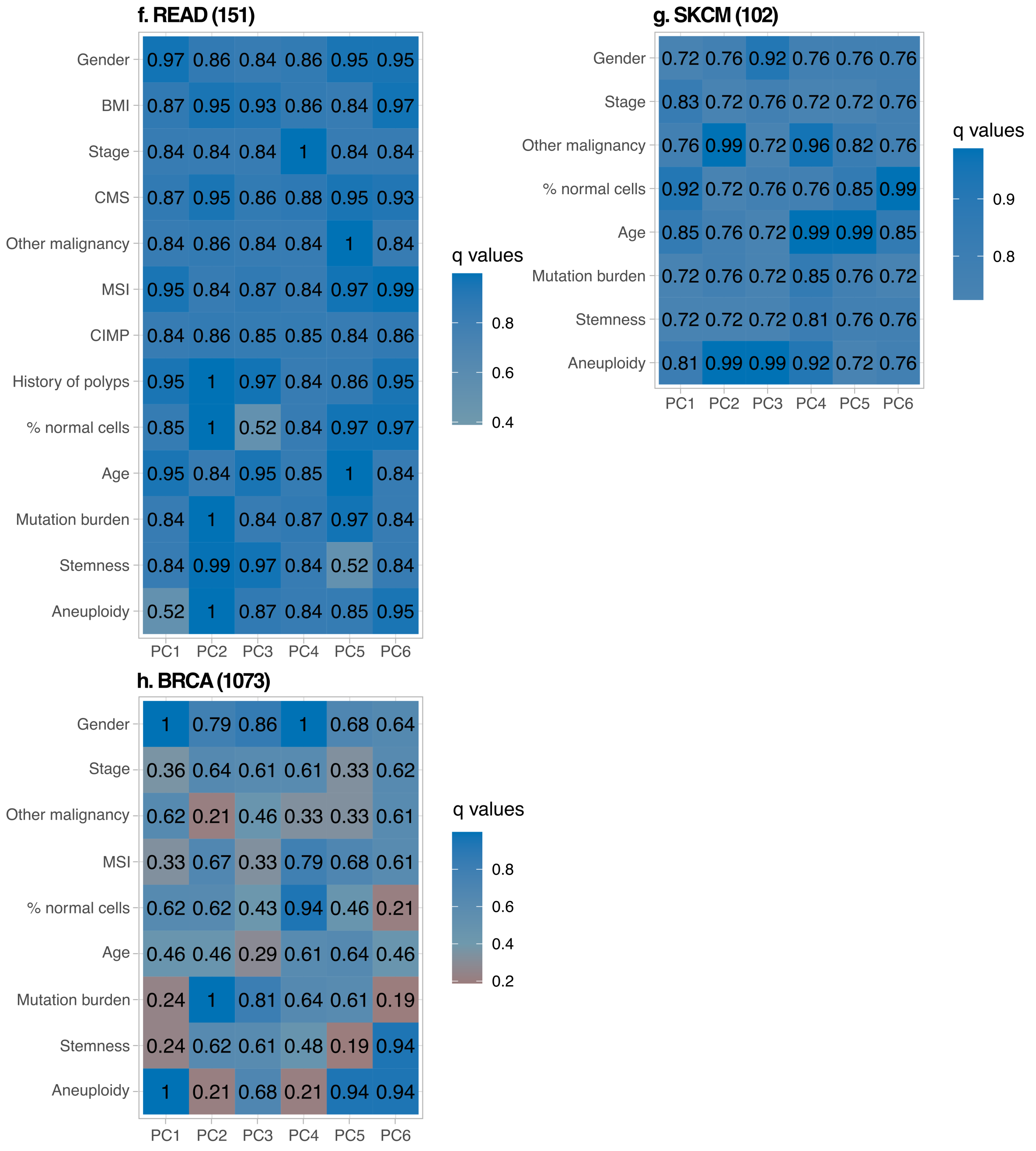

Fig S5. Heatmaps of the *q* values of the associations and correlations between the most important bacterial species and the clinical properties from the metadata of (a) glioblastoma multiforme (GBM), (b) lung adenocarcinoma (LUAD), (c) lung squamous cell carcinoma (LUSC), (d) head and neck squamous cell neoplasms (HNSC), (e) ovarian serous cystadenocarcinoma (OV), (f) rectum adenocarcinoma (READ), (g) skin cutaneous melanoma (SKCM) and (h) breast invasive carcinoma (BRCA). No batch correction has been applied on GBM bacteria quantification, while the other tissues underwent plate ID correction. CIMP, CpG methylation phenotype; MSI, mi-

crosatellite instability; CMS, consensus molecular subtype; BMI, body mass index. Number of samples analysed in brackets.

Sambruni et al.

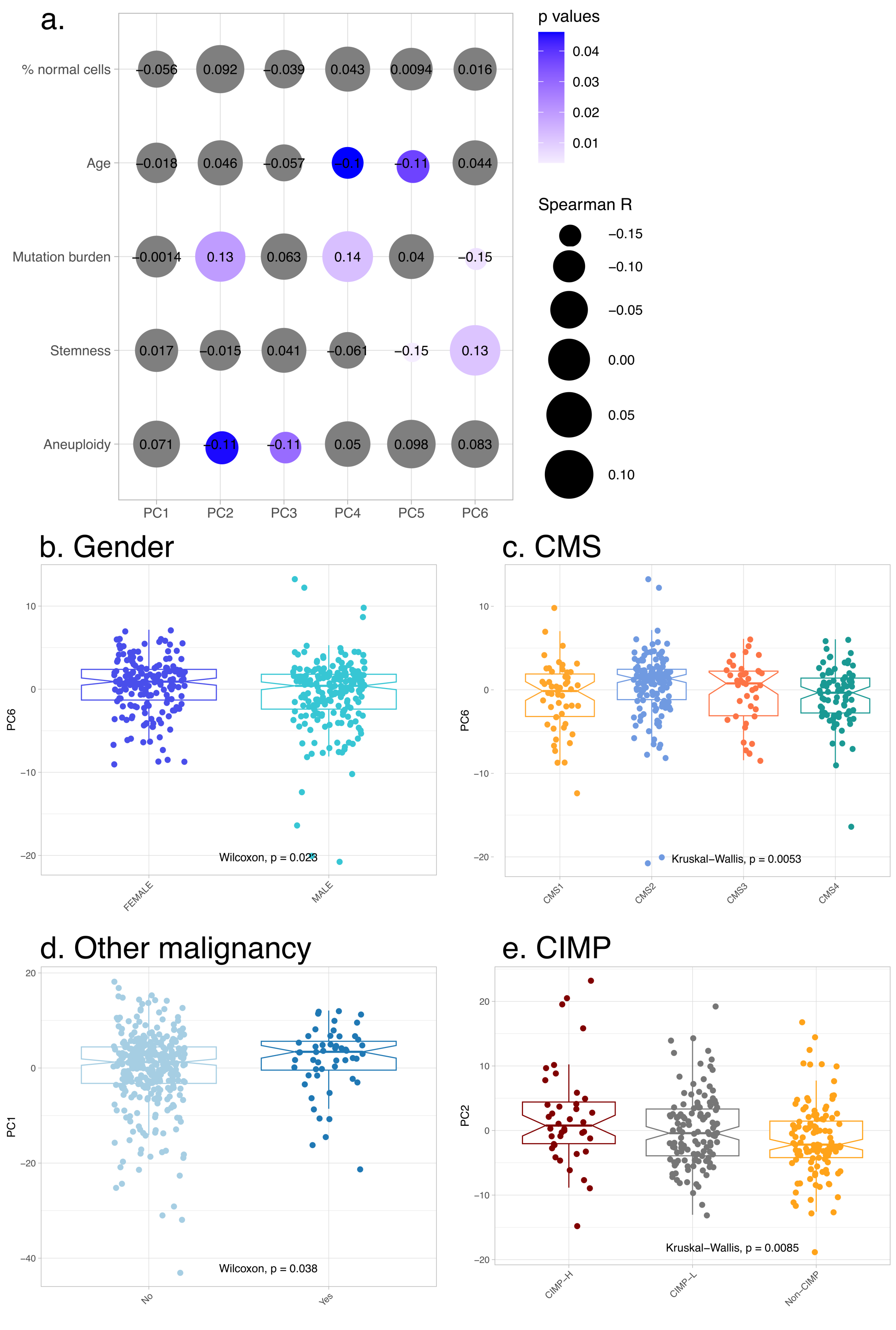

f. CIMP

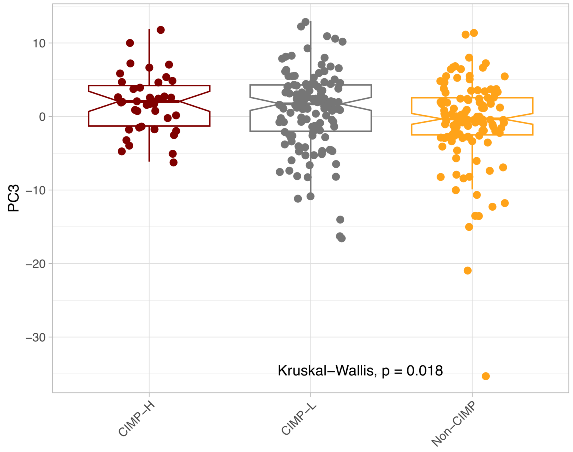

g. CIMP

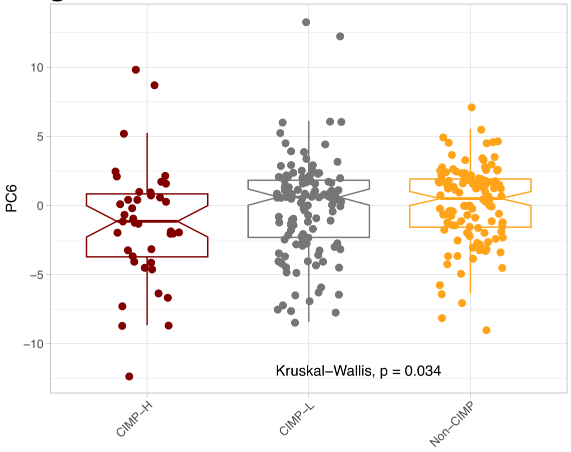

h. Side

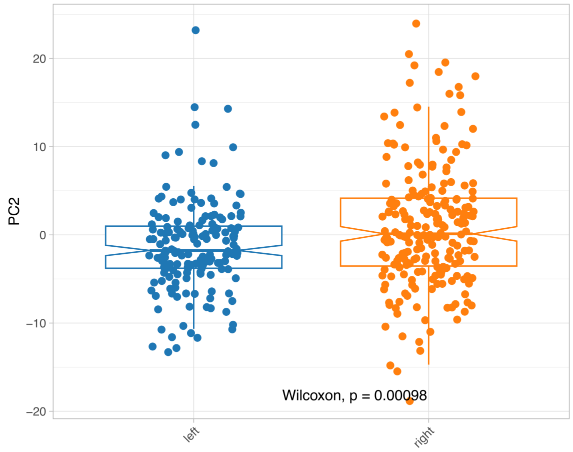

i. Side

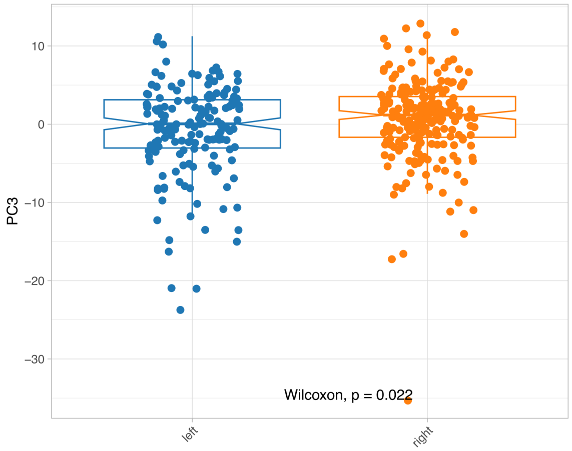

j. MSI

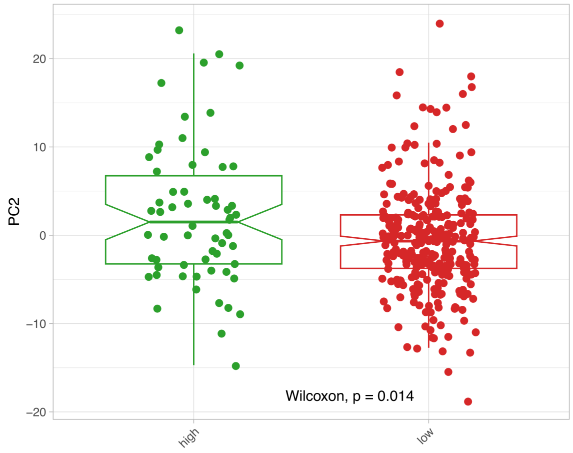

k. MSI

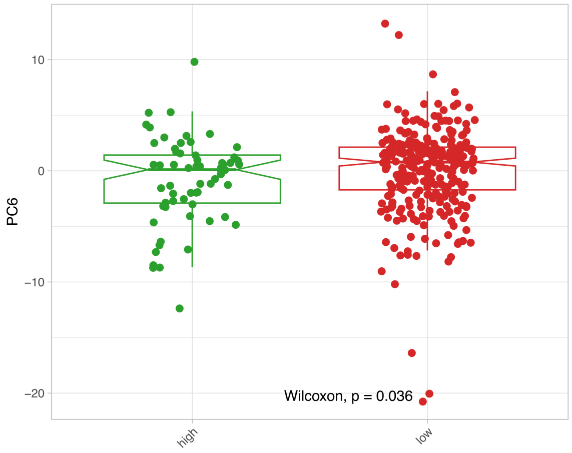

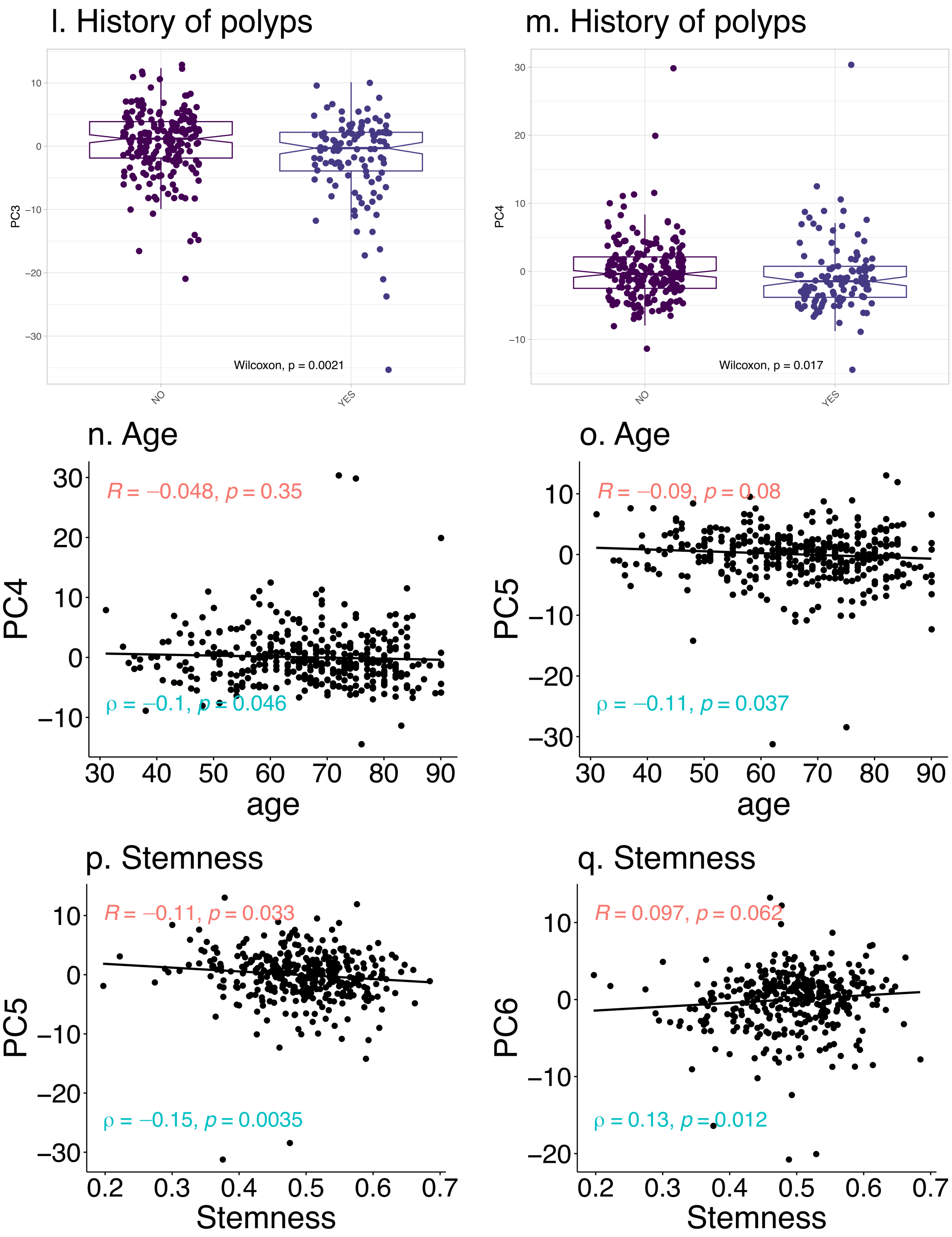
l. History of polyps
PC3
NO
YES
Wilcoxon, p = 0.0021
m. History of polyps
PC4
NO
YES
Wilcoxon, p = 0.017
n. Age
PC4
age
R = −0.048, p = 0.35
ρ = −0.1, p = 0.046
o. Age
PC5
age
R = −0.09, p = 0.08
ρ = −0.11, p = 0.037
p. Stemness
PC5
Stemness
R = −0.11, p = 0.033
ρ = −0.15, p = 0.0035
q. Stemness
PC6
Stemness
R = 0.097, p = 0.062
ρ = 0.13, p = 0.012

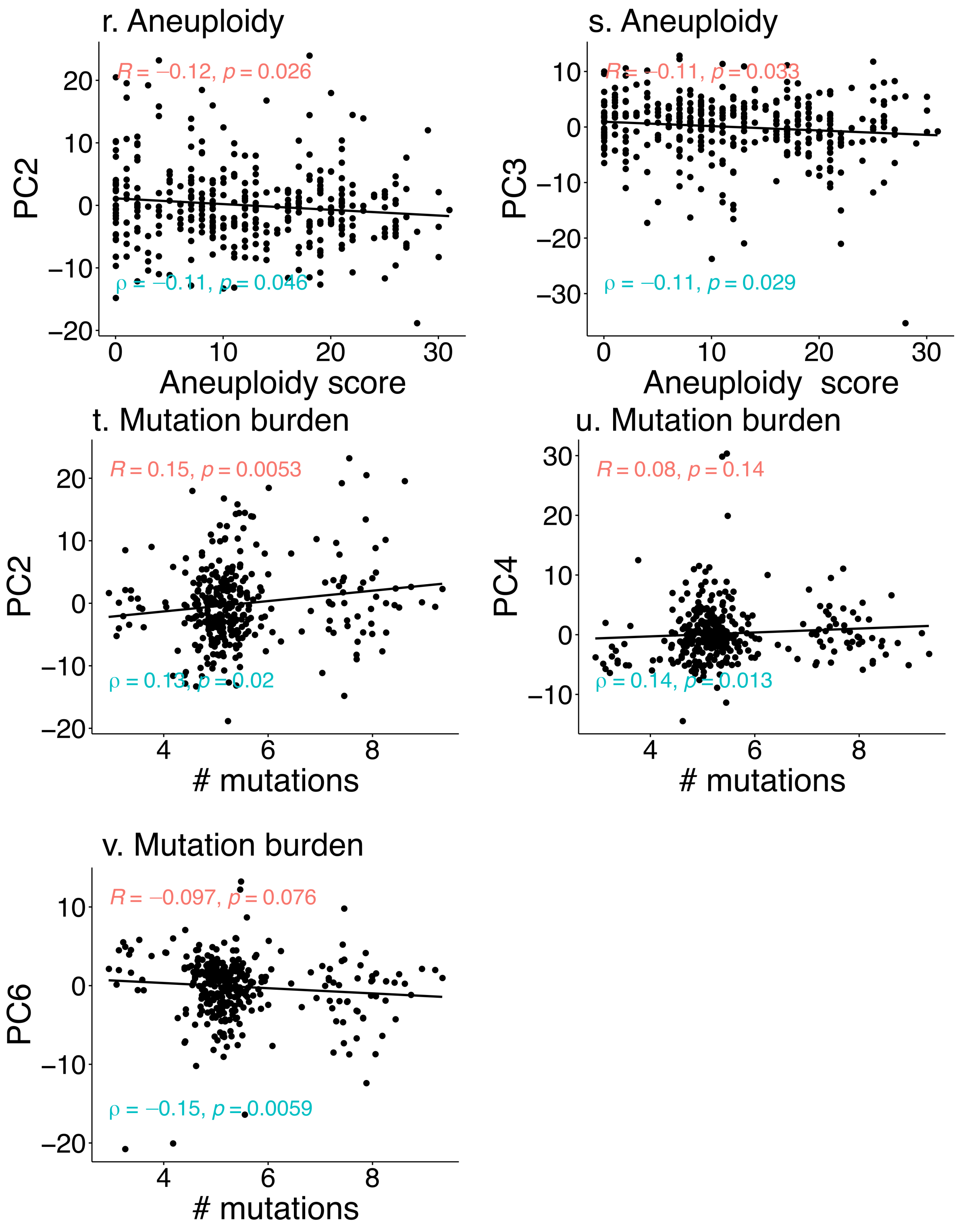
r. Aneuploidy
R = −0.12, p = 0.026
ρ = −0.11, p = 0.046
PC2
Aneuploidy score
s. Aneuploidy
R = −0.11, p = 0.033
ρ = −0.11, p = 0.029
PC3
Aneuploidy score
t. Mutation burden
R = 0.15, p = 0.0053
ρ = 0.13, p = 0.02
PC2
mutations
u. Mutation burden
R = 0.08, p = 0.14
ρ = 0.14, p = 0.013
PC4
mutations
v. Mutation burden
R = −0.097, p = 0.076
ρ = −0.15, p = 0.0059
PC6
mutations

Fig S6. Bubble plot showing the *p* values and the Spearman R values of the correlation between the first six principal components (PCs) of colon adenocarcinoma (COAD) primary tumour principal component analysis and the continuous clinical properties (a). Boxplots and scatter plots of the PCs coordinates associated with clinical properties in COAD: (b) gender, (c) consensus molecular subtype (CMS), (d) other malignancy, (e,f,g) CpG methylation phenotype (CIMP), (h,i) side, (j,k) microsatellite instability (MSI), (l,m) history of polyps, (n,o) age, (p,q) stemness, (r,s) aneuploidy, (t,u,v) mutation burden. Spearman test (light blue), Pearson test (pink).

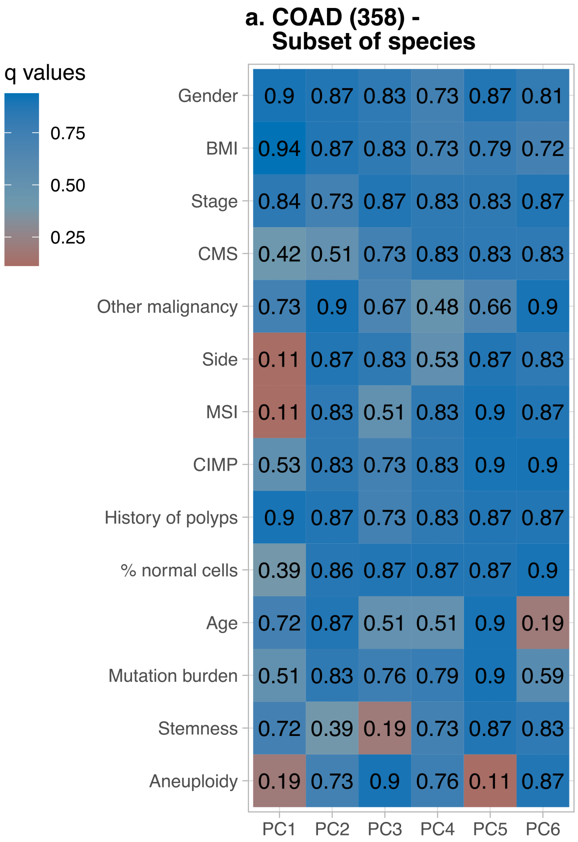

Fig S7. Heatmaps of the *q* values of the associations and correlation between the first six principal components (PCs) of the principal component analysis on a strict subset of abundant, tissue-specific and reduced in technical variability species from the reconstructed microbiome of the associations between clinical tumoural properties of colon adenocarcinoma (COAD) primary tumours. CIMP, CpG methylation phenotype; MSI, microsatellite instability; BMI, body mass index. Number of samples analysed in brackets.

Sambruni et al.

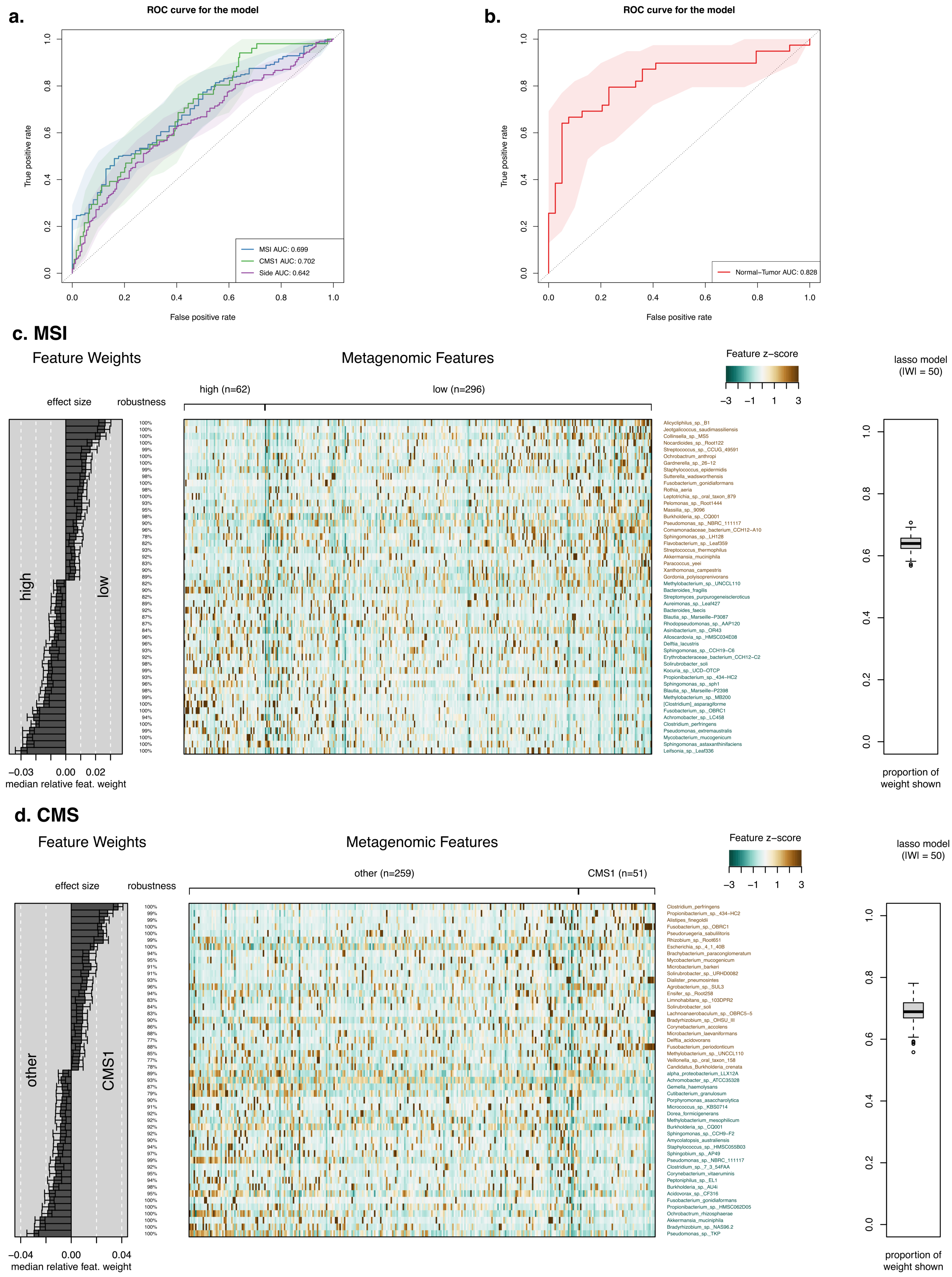

e. Side

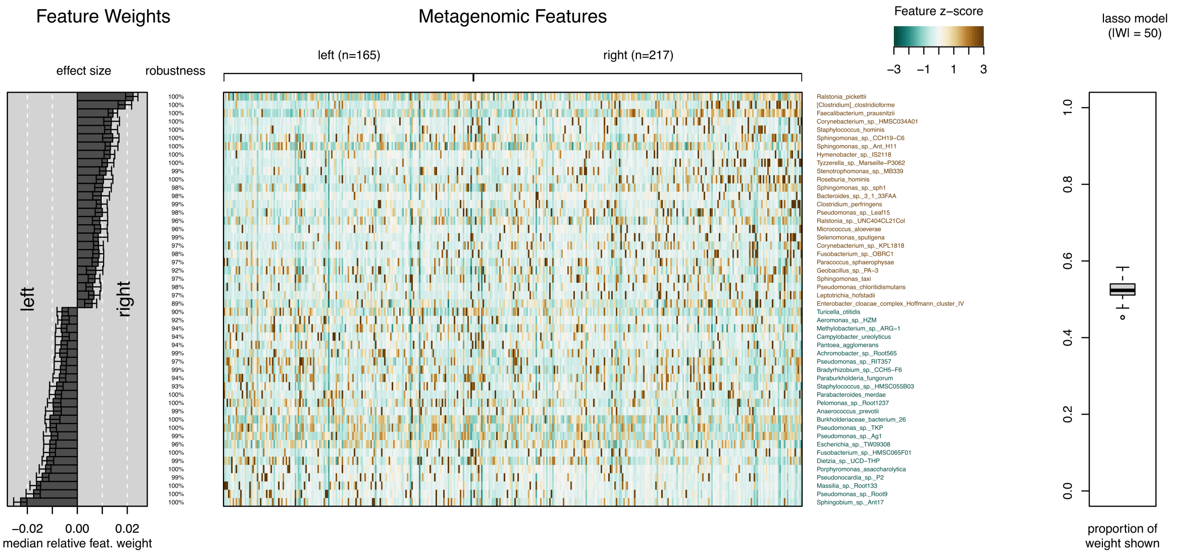

f. Status (normal vs tumour)

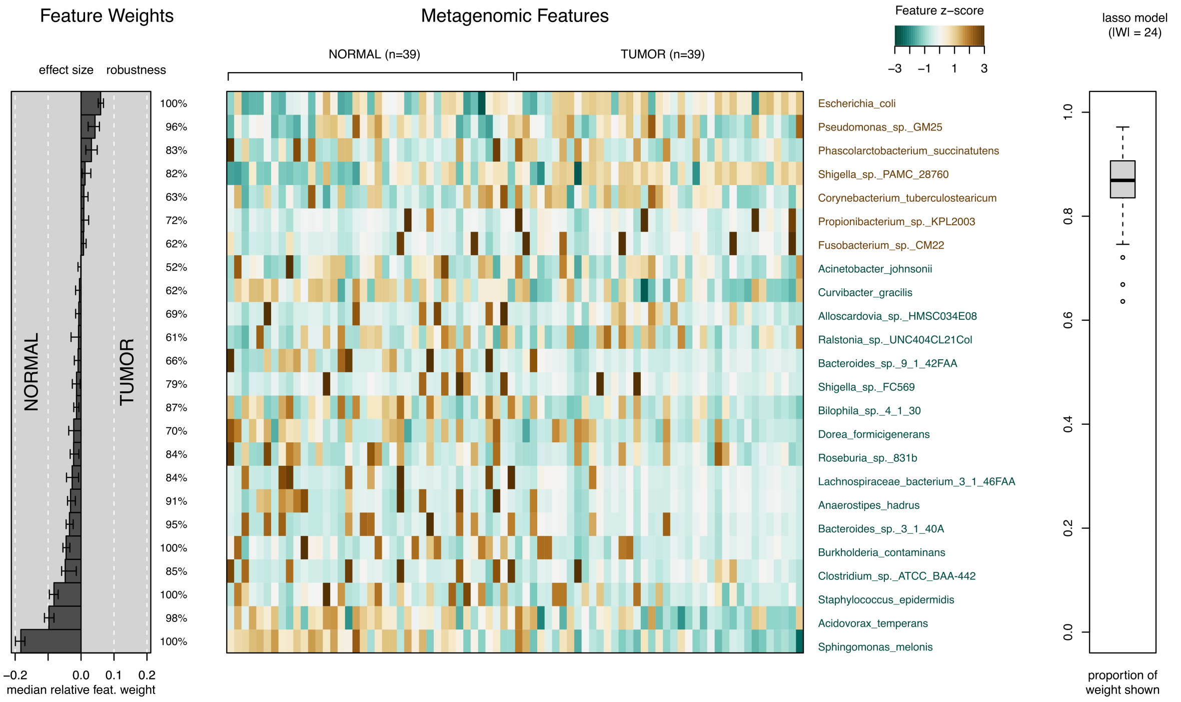

Fig S8. Receiver operating characteristic (ROC) curves for the ridge model to classify (a) the clinical properties (side, microsatellite instability (MSI) and consensus molecular subtypes (CMS)) and (b) the status of the samples (normal vs tumour). Metagenomic features selected by the ridge models to classify (c) the MSI, (d) the CMS1, (e) the side and (f) the status. AUC, area under the ROC curve.

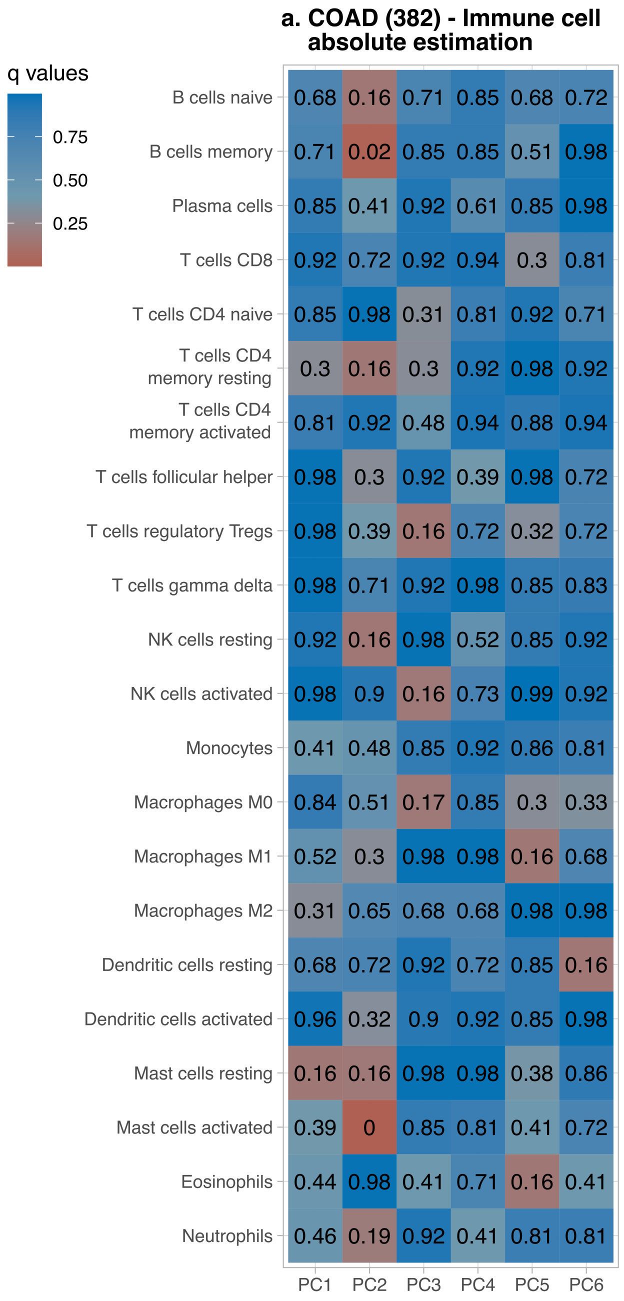

Fig S9. Heatmaps of the *q* values of the associations and correlation between the first six principal components (PCs) of the principal component analysis from the reconstructed microbiome of the associations between the absolute immune cell estimations of colon adenocarcinoma (COAD) primary tumours (a). NK, natural killer. Number of samples analysed in brackets.

Sambruni et al.

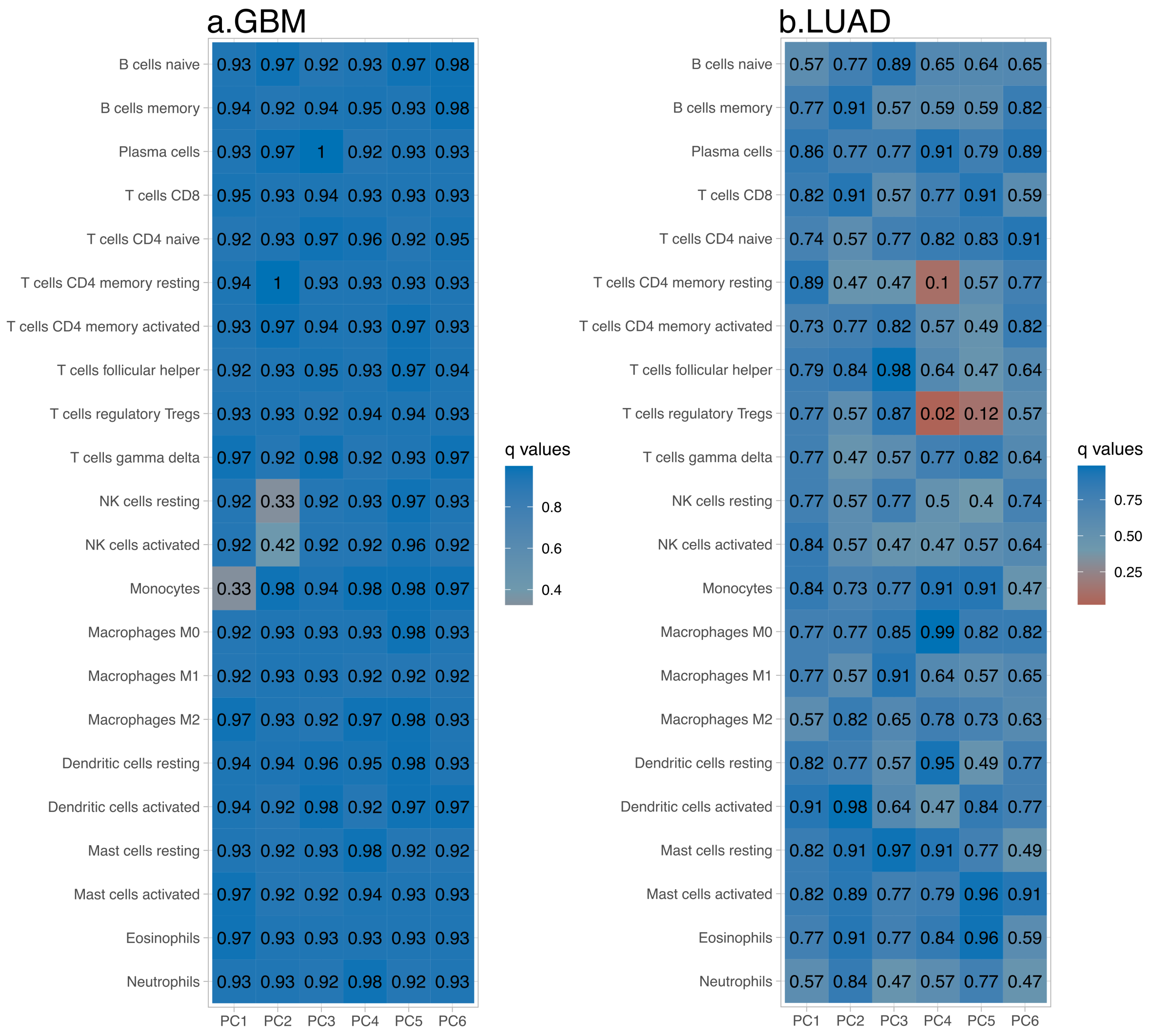

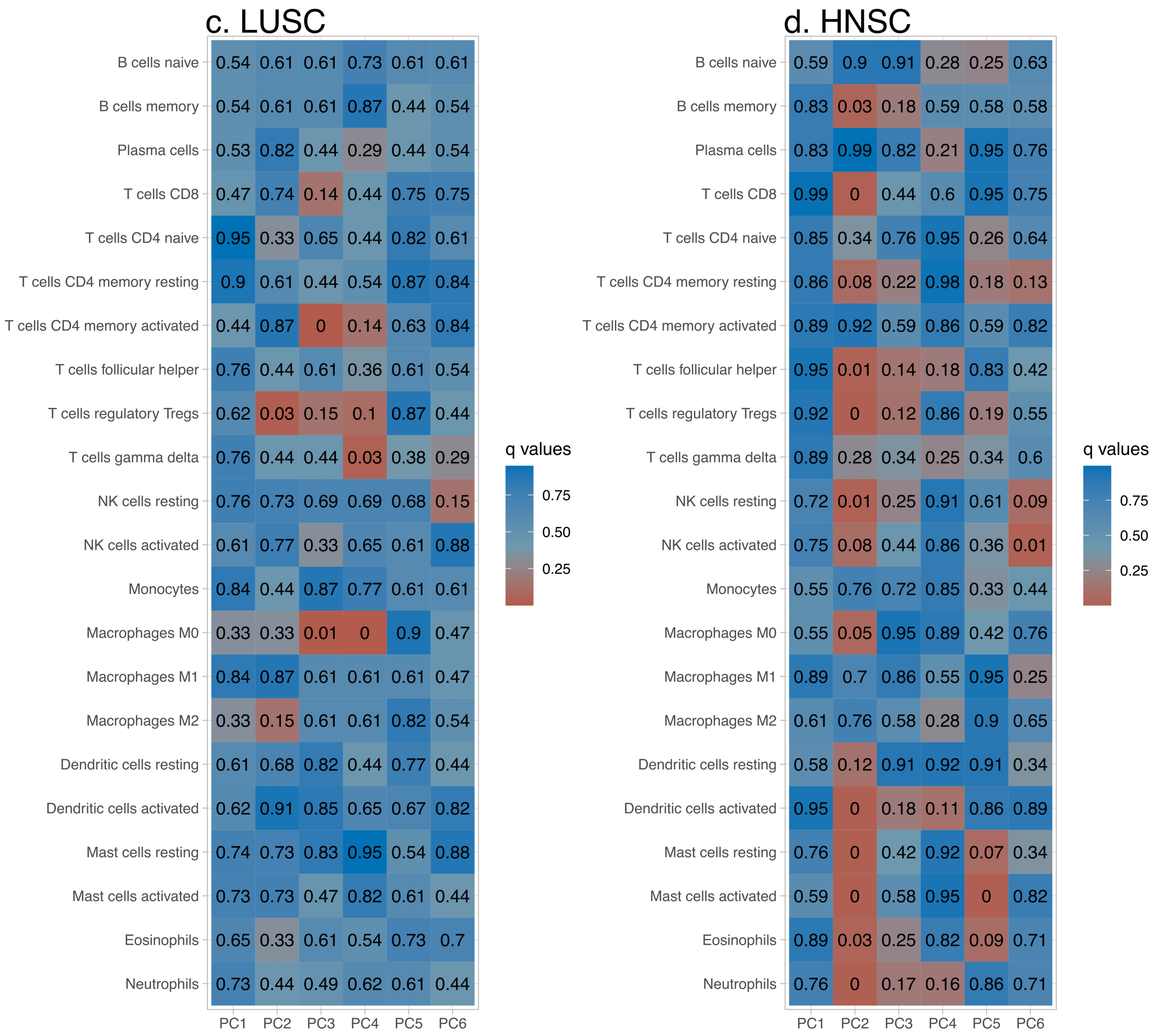
c. LUSC
B cells naive 0.54 0.61 0.61 0.73 0.61 0.61
B cells memory 0.54 0.61 0.61 0.87 0.44 0.54
Plasma cells 0.53 0.82 0.44 0.29 0.44 0.54
T cells CD8 0.47 0.74 0.14 0.44 0.75 0.75
T cells CD4 naive 0.95 0.33 0.65 0.44 0.82 0.61
T cells CD4 memory resting 0.9 0.61 0.44 0.54 0.87 0.84
T cells CD4 memory activated 0.44 0.87 0 0.14 0.63 0.84
T cells follicular helper 0.76 0.44 0.61 0.36 0.61 0.54
T cells regulatory Tregs 0.62 0.03 0.15 0.1 0.87 0.44
T cells gamma delta 0.76 0.44 0.44 0.03 0.38 0.29
NK cells resting 0.76 0.73 0.69 0.69 0.68 0.15
NK cells activated 0.61 0.77 0.33 0.65 0.61 0.88
Monocytes 0.84 0.44 0.87 0.77 0.61 0.61
Macrophages M0 0.33 0.33 0.01 0 0.9 0.47
Macrophages M1 0.84 0.87 0.61 0.61 0.61 0.47
Macrophages M2 0.33 0.15 0.61 0.61 0.82 0.54
Dendritic cells resting 0.61 0.68 0.82 0.44 0.77 0.44
Dendritic cells activated 0.62 0.91 0.85 0.65 0.67 0.82
Mast cells resting 0.74 0.73 0.83 0.95 0.54 0.88
Mast cells activated 0.73 0.73 0.47 0.82 0.61 0.44
Eosinophils 0.65 0.33 0.61 0.54 0.73 0.7
Neutrophils 0.73 0.44 0.49 0.62 0.61 0.44
PC1 PC2 PC3 PC4 PC5 PC6
q values
0.75
0.50
0.25
d. HNSC
B cells naive 0.59 0.9 0.91 0.28 0.25 0.63
B cells memory 0.83 0.03 0.18 0.59 0.58 0.58
Plasma cells 0.83 0.99 0.82 0.21 0.95 0.76
T cells CD8 0.99 0 0.44 0.6 0.95 0.75
T cells CD4 naive 0.85 0.34 0.76 0.95 0.26 0.64
T cells CD4 memory resting 0.86 0.08 0.22 0.98 0.18 0.13
T cells CD4 memory activated 0.89 0.92 0.59 0.86 0.59 0.82
T cells follicular helper 0.95 0.01 0.14 0.18 0.83 0.42
T cells regulatory Tregs 0.92 0 0.12 0.86 0.19 0.55
T cells gamma delta 0.89 0.28 0.34 0.25 0.34 0.6
NK cells resting 0.72 0.01 0.25 0.91 0.61 0.09
NK cells activated 0.75 0.08 0.44 0.86 0.36 0.01
Monocytes 0.55 0.76 0.72 0.85 0.33 0.44
Macrophages M0 0.55 0.05 0.95 0.89 0.42 0.76
Macrophages M1 0.89 0.7 0.86 0.55 0.95 0.25
Macrophages M2 0.61 0.76 0.58 0.28 0.9 0.65
Dendritic cells resting 0.58 0.12 0.91 0.92 0.91 0.34
Dendritic cells activated 0.95 0 0.18 0.11 0.86 0.89
Mast cells resting 0.76 0 0.42 0.92 0.07 0.34
Mast cells activated 0.59 0 0.58 0.95 0 0.82
Eosinophils 0.89 0.03 0.25 0.82 0.09 0.71
Neutrophils 0.76 0 0.17 0.16 0.86 0.71
PC1 PC2 PC3 PC4 PC5 PC6
q values
0.75
0.50
0.25

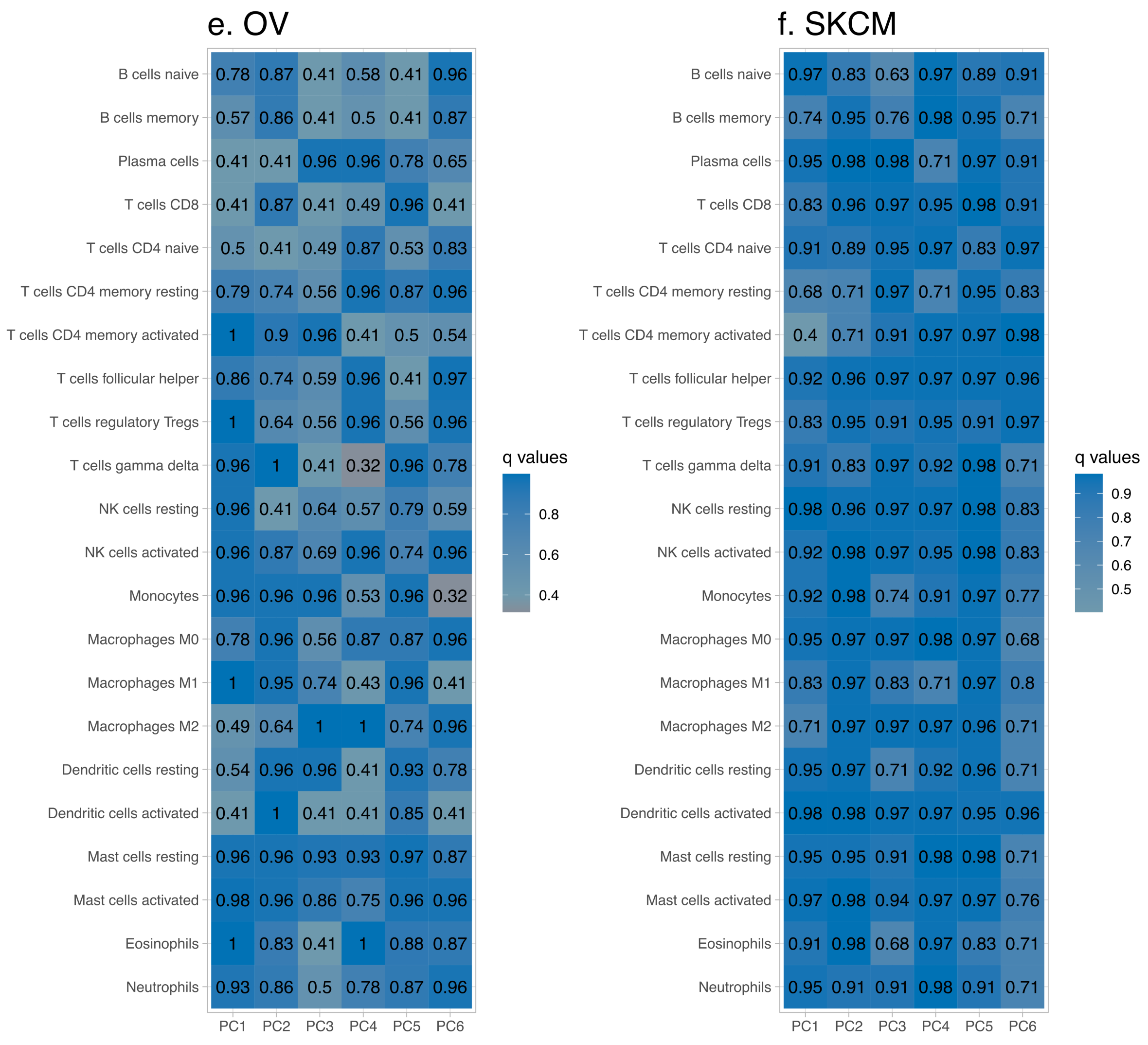
e. OV
B cells naive 0.78 0.87 0.41 0.58 0.41 0.96
B cells memory 0.57 0.86 0.41 0.5 0.41 0.87
Plasma cells 0.41 0.41 0.96 0.96 0.78 0.65
T cells CD8 0.41 0.87 0.41 0.49 0.96 0.41
T cells CD4 naive 0.5 0.41 0.49 0.87 0.53 0.83
T cells CD4 memory resting 0.79 0.74 0.56 0.96 0.87 0.96
T cells CD4 memory activated 1 0.9 0.96 0.41 0.5 0.54
T cells follicular helper 0.86 0.74 0.59 0.96 0.41 0.97
T cells regulatory Tregs 1 0.64 0.56 0.96 0.56 0.96
T cells gamma delta 0.96 1 0.41 0.32 0.96 0.78
NK cells resting 0.96 0.41 0.64 0.57 0.79 0.59
NK cells activated 0.96 0.87 0.69 0.96 0.74 0.96
Monocytes 0.96 0.96 0.96 0.53 0.96 0.32
Macrophages M0 0.78 0.96 0.56 0.87 0.87 0.96
Macrophages M1 1 0.95 0.74 0.43 0.96 0.41
Macrophages M2 0.49 0.64 1 1 0.74 0.96
Dendritic cells resting 0.54 0.96 0.96 0.41 0.93 0.78
Dendritic cells activated 0.41 1 0.41 0.41 0.85 0.41
Mast cells resting 0.96 0.96 0.93 0.93 0.97 0.87
Mast cells activated 0.98 0.96 0.86 0.75 0.96 0.96
Eosinophils 1 0.83 0.41 1 0.88 0.87
Neutrophils 0.93 0.86 0.5 0.78 0.87 0.96
PC1 PC2 PC3 PC4 PC5 PC6
q values
0.8
0.6
0.4
f. SKCM
B cells naive 0.97 0.83 0.63 0.97 0.89 0.91
B cells memory 0.74 0.95 0.76 0.98 0.95 0.71
Plasma cells 0.95 0.98 0.98 0.71 0.97 0.91
T cells CD8 0.83 0.96 0.97 0.95 0.98 0.91
T cells CD4 naive 0.91 0.89 0.95 0.97 0.83 0.97
T cells CD4 memory resting 0.68 0.71 0.97 0.71 0.95 0.83
T cells CD4 memory activated 0.4 0.71 0.91 0.97 0.97 0.98
T cells follicular helper 0.92 0.96 0.97 0.97 0.97 0.96
T cells regulatory Tregs 0.83 0.95 0.91 0.95 0.91 0.97
T cells gamma delta 0.91 0.83 0.97 0.92 0.98 0.71
NK cells resting 0.98 0.96 0.97 0.97 0.98 0.83
NK cells activated 0.92 0.98 0.97 0.95 0.98 0.83
Monocytes 0.92 0.98 0.74 0.91 0.97 0.77
Macrophages M0 0.95 0.97 0.97 0.98 0.97 0.68
Macrophages M1 0.83 0.97 0.83 0.71 0.97 0.8
Macrophages M2 0.71 0.97 0.97 0.97 0.96 0.71
Dendritic cells resting 0.95 0.97 0.71 0.92 0.96 0.71
Dendritic cells activated 0.98 0.98 0.97 0.97 0.95 0.96
Mast cells resting 0.95 0.95 0.91 0.98 0.98 0.71
Mast cells activated 0.97 0.98 0.94 0.97 0.97 0.76
Eosinophils 0.91 0.98 0.68 0.97 0.83 0.71
Neutrophils 0.95 0.91 0.91 0.98 0.91 0.71
PC1 PC2 PC3 PC4 PC5 PC6
q values
0.9
0.8
0.7
0.6
0.5

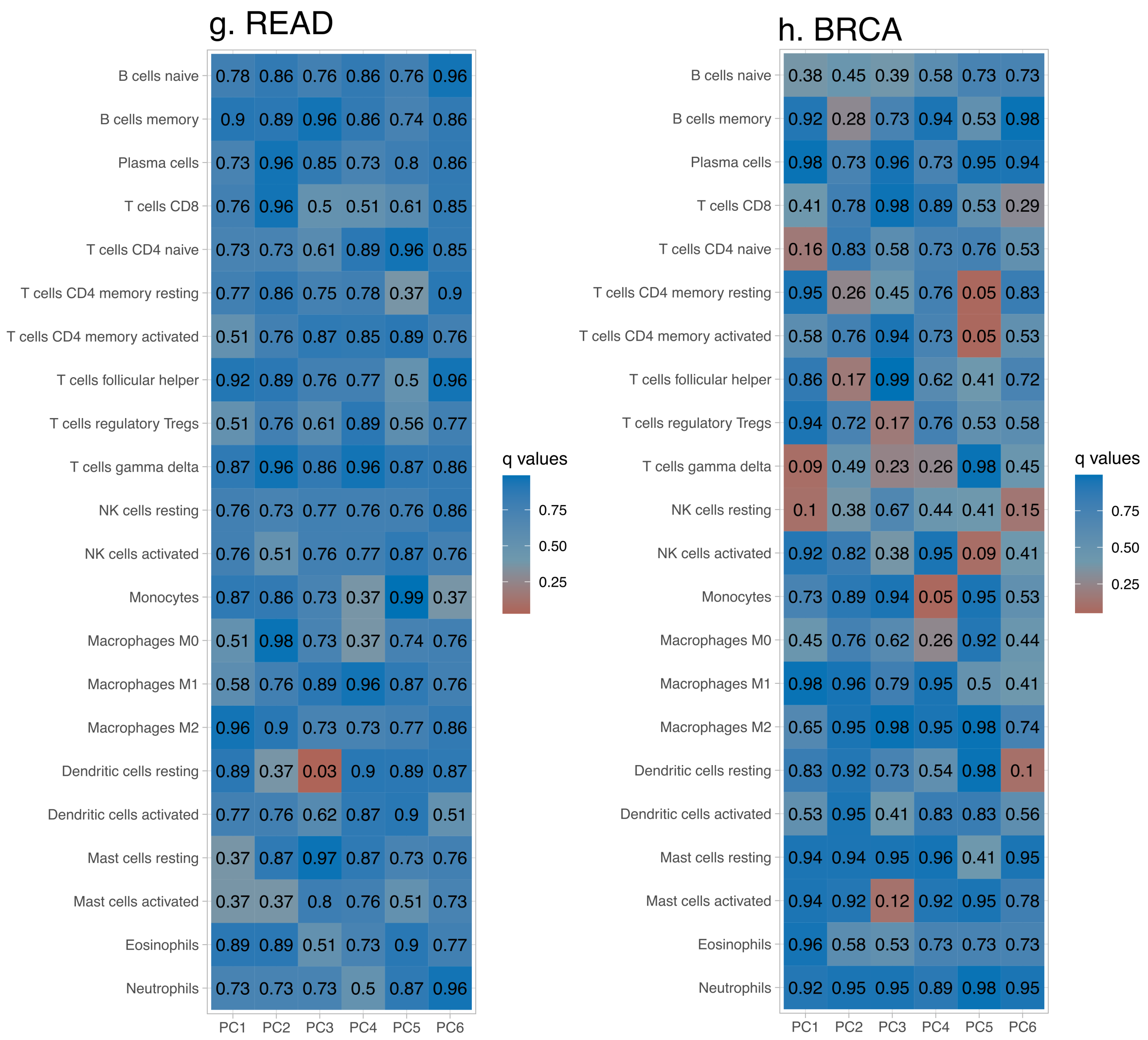

Fig S10. Heatmaps of the *q* values of the associations and correlation between the first six principal components (PCs) of the principal component analysis on the reconstructed microbiome with relative immune cell estimation from CIBERSORTx of (a) glioblastoma multiforme (GBM), (b) lung adenocarcinoma (LUAD), (c) lung squamous cell carcinoma (LUSC), (d) head and neck squamous cell neoplasms (HNSC), (e) ovarian serous cystadenocarcinoma (OV), (f) skin cutaneous melanoma (SKCM), (g) rectum adenocarcinoma (READ) and (h) breast invasive carcinoma (BRCA) samples. NK, natural killer.

a. COAD (382) - Mutation status

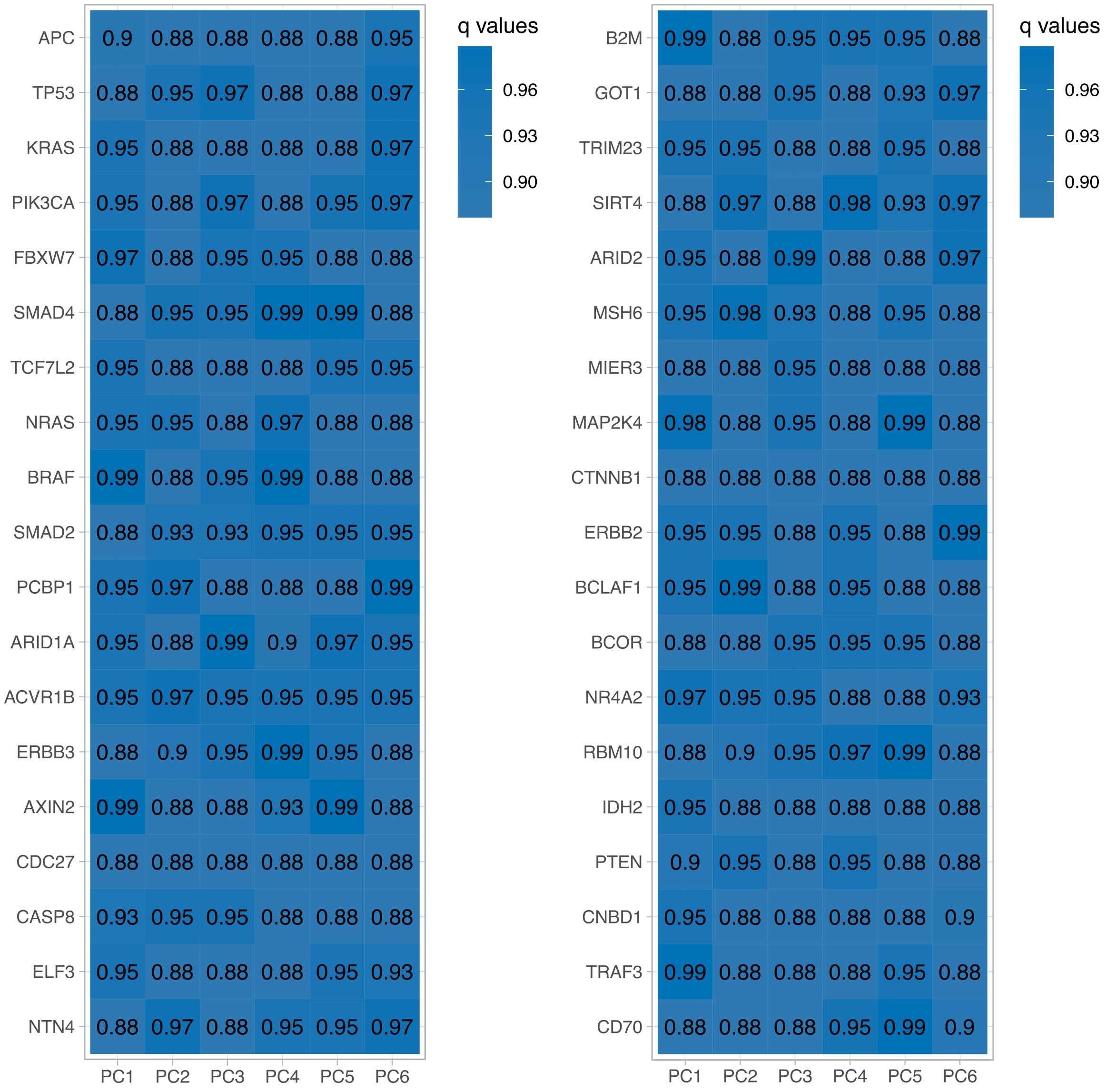

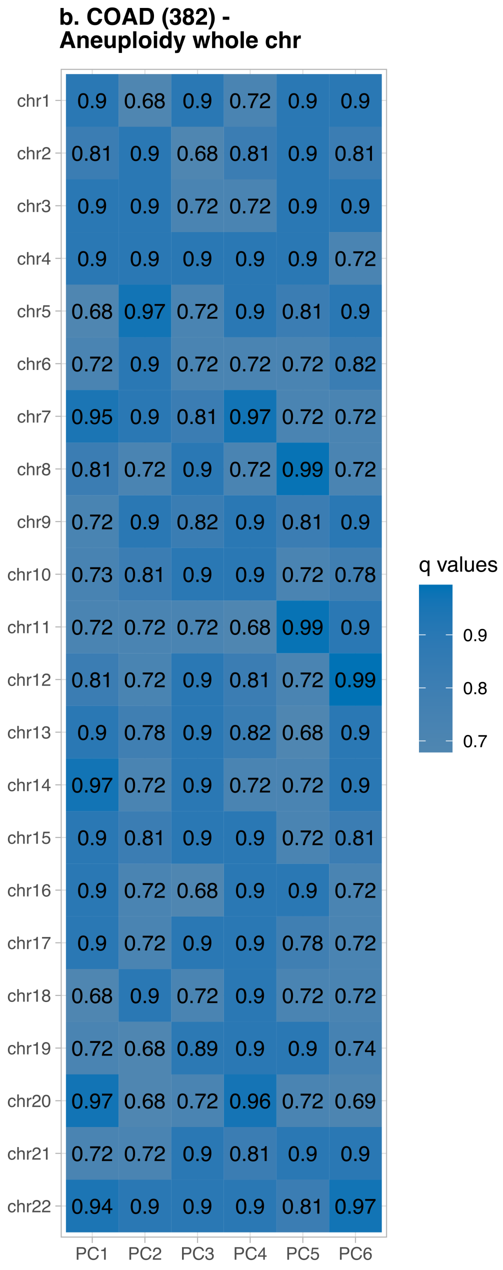

Fig S11. Heatmaps of the *q* values of the associations and correlation between the first six principal components (PCs) of the principal component analysis on the reconstructed microbiome with (a) the mutation status of colon adenocarcinoma (COAD) driver genes and with (b) the aneuploidy whole chromosome (chr) status of COAD. Number of samples analysed in brackets.

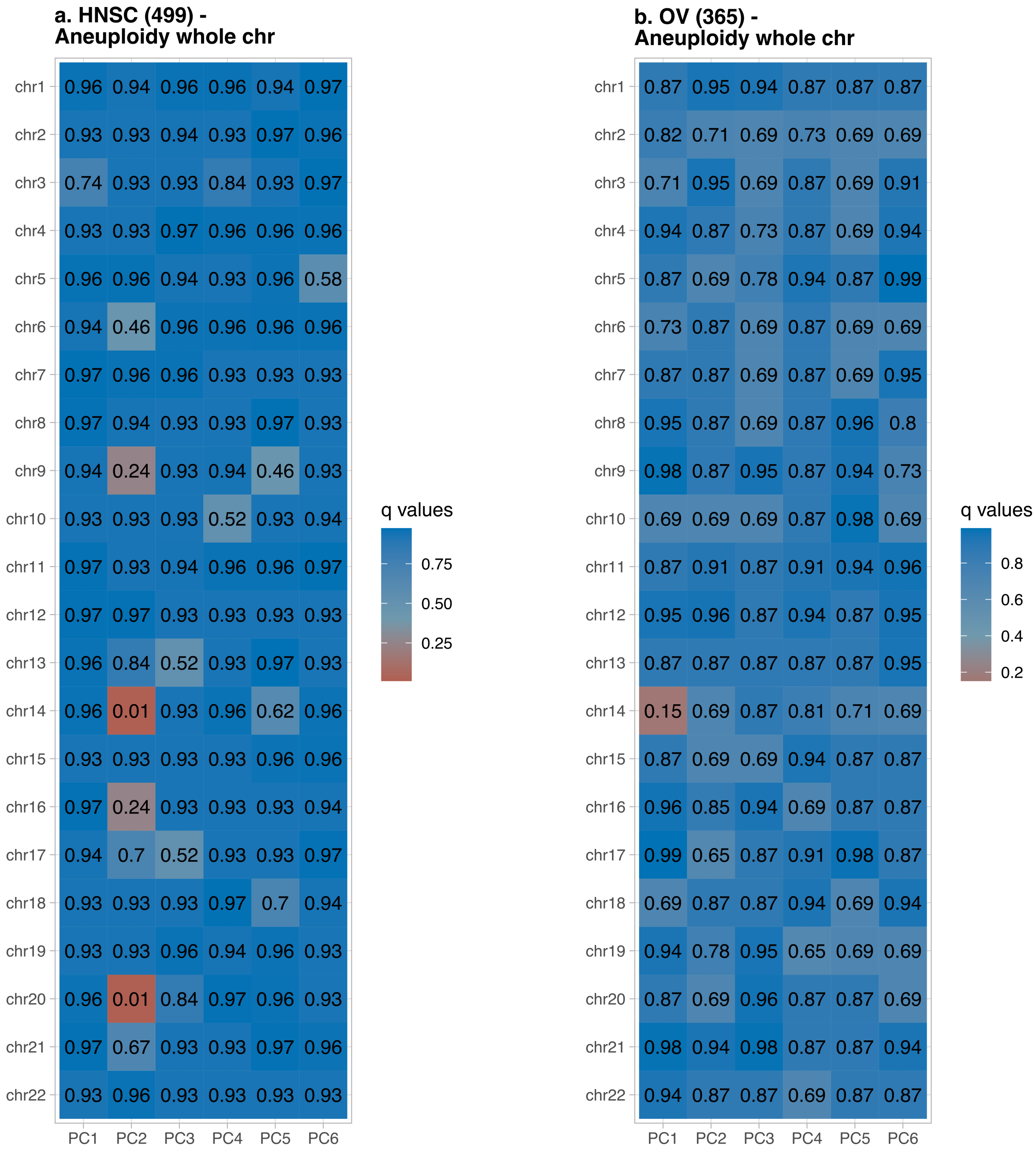
a. HNSC (499) -
Aneuploidy whole chr
chr1 0.96 0.94 0.96 0.96 0.94 0.97
chr2 0.93 0.93 0.94 0.93 0.97 0.96
chr3 0.74 0.93 0.93 0.84 0.93 0.97
chr4 0.93 0.93 0.97 0.96 0.96 0.96
chr5 0.96 0.96 0.94 0.93 0.96 0.58
chr6 0.94 0.46 0.96 0.96 0.96 0.96
chr7 0.97 0.96 0.96 0.93 0.93 0.93
chr8 0.97 0.94 0.93 0.93 0.97 0.93
chr9 0.94 0.24 0.93 0.94 0.46 0.93
chr10 0.93 0.93 0.93 0.52 0.93 0.94
chr11 0.97 0.93 0.94 0.96 0.96 0.97
chr12 0.97 0.97 0.93 0.93 0.93 0.93
chr13 0.96 0.84 0.52 0.93 0.97 0.93
chr14 0.96 0.01 0.93 0.96 0.62 0.96
chr15 0.93 0.93 0.93 0.93 0.96 0.96
chr16 0.97 0.24 0.93 0.93 0.93 0.94
chr17 0.94 0.7 0.52 0.93 0.93 0.97
chr18 0.93 0.93 0.93 0.97 0.7 0.94
chr19 0.93 0.93 0.96 0.94 0.96 0.93
chr20 0.96 0.01 0.84 0.97 0.96 0.93
chr21 0.97 0.67 0.93 0.93 0.97 0.96
chr22 0.93 0.96 0.93 0.93 0.93 0.93
PC1 PC2 PC3 PC4 PC5 PC6
q values
0.75
0.50
0.25
b. OV (365) -
Aneuploidy whole chr
chr1 0.87 0.95 0.94 0.87 0.87 0.87
chr2 0.82 0.71 0.69 0.73 0.69 0.69
chr3 0.71 0.95 0.69 0.87 0.69 0.91
chr4 0.94 0.87 0.73 0.87 0.69 0.94
chr5 0.87 0.69 0.78 0.94 0.87 0.99
chr6 0.73 0.87 0.69 0.87 0.69 0.69
chr7 0.87 0.87 0.69 0.87 0.69 0.95
chr8 0.95 0.87 0.69 0.87 0.96 0.8
chr9 0.98 0.87 0.95 0.87 0.94 0.73
chr10 0.69 0.69 0.69 0.87 0.98 0.69
chr11 0.87 0.91 0.87 0.91 0.94 0.96
chr12 0.95 0.96 0.87 0.94 0.87 0.95
chr13 0.87 0.87 0.87 0.87 0.87 0.95
chr14 0.15 0.69 0.87 0.81 0.71 0.69
chr15 0.87 0.69 0.69 0.94 0.87 0.87
chr16 0.96 0.85 0.94 0.69 0.87 0.87
chr17 0.99 0.65 0.87 0.91 0.98 0.87
chr18 0.69 0.87 0.87 0.94 0.69 0.94
chr19 0.94 0.78 0.95 0.65 0.69 0.69
chr20 0.87 0.69 0.96 0.87 0.87 0.69
chr21 0.98 0.94 0.98 0.87 0.87 0.94
chr22 0.94 0.87 0.87 0.69 0.87 0.87
PC1 PC2 PC3 PC4 PC5 PC6
q values
0.8
0.6
0.4
0.2

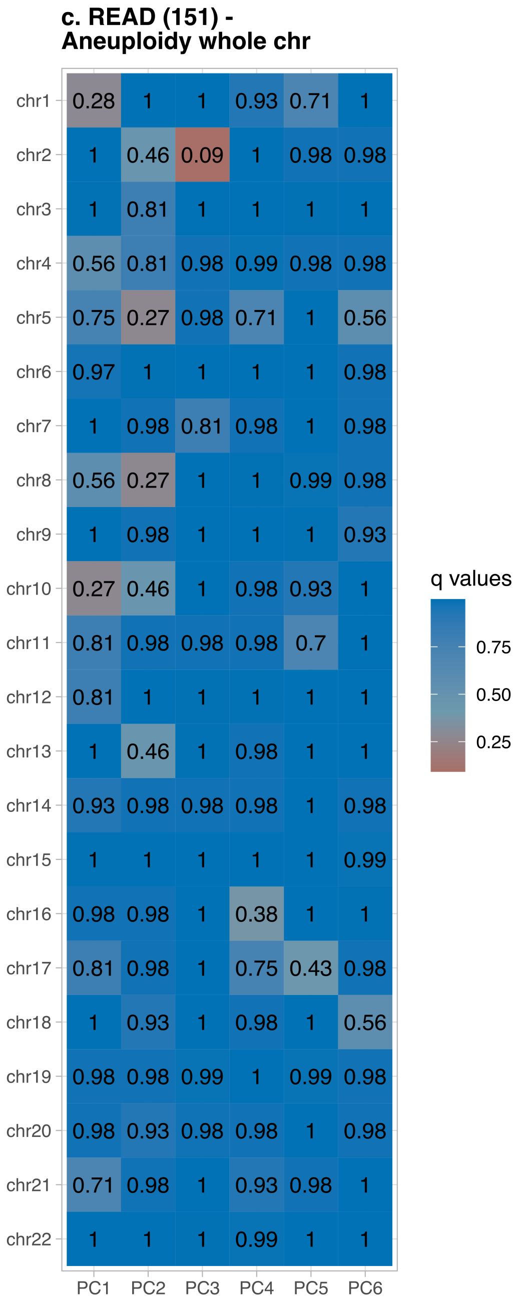

Fig S12. Heatmaps of the *q* values of the associations and correlation between the first six principal components (PCs) of the principal component analysis on the reconstructed microbiome with the aneuploidy whole chromosome (chr) status of (a) head and neck squamous cell neoplasms (HNSC), (b) ovarian serous cystadenocarcinoma (OV) and (c) rectum adenocarcinoma (READ) samples. Number of samples analysed in brackets.

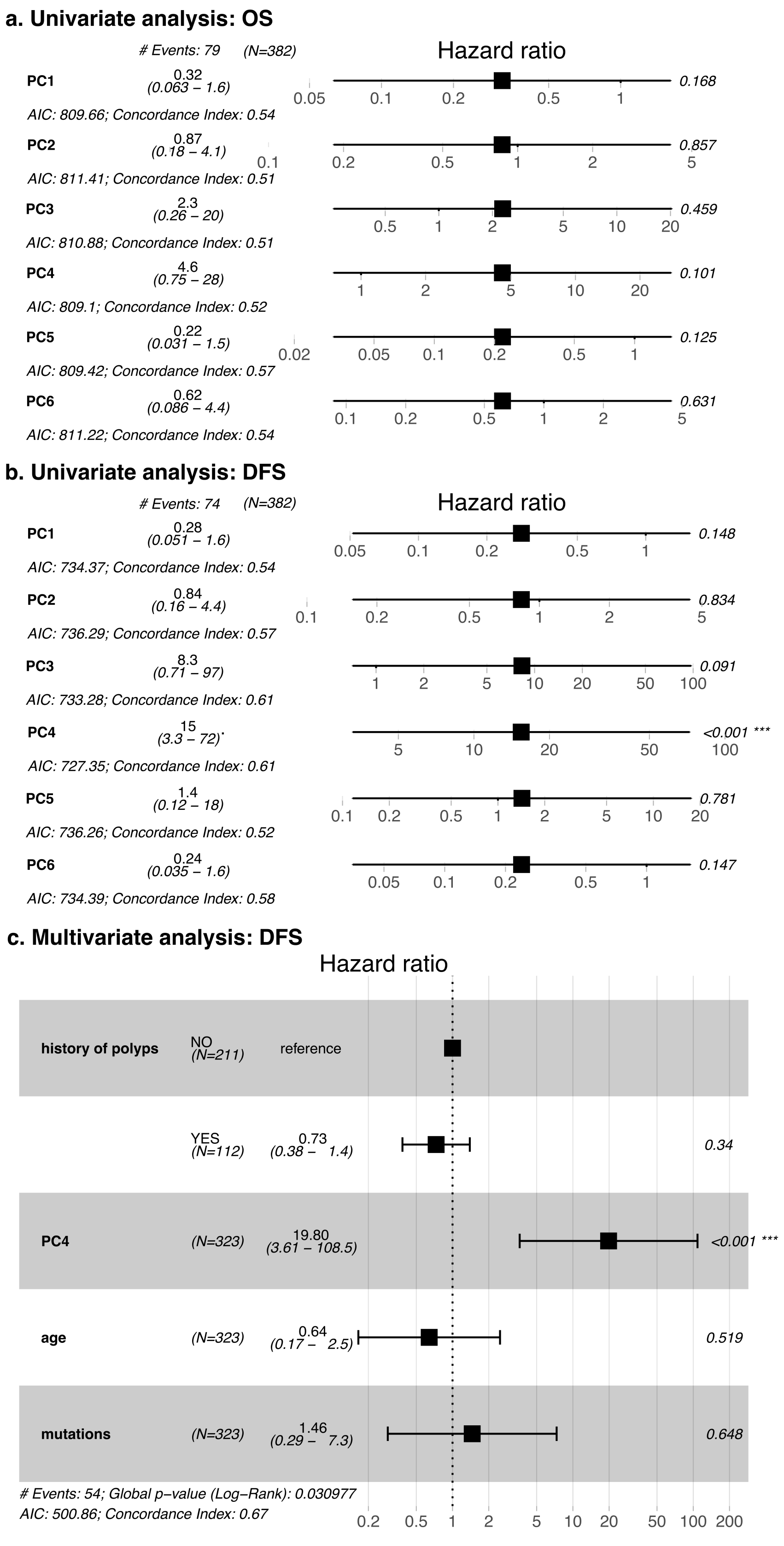
a. Univariate analysis: OS
Events: 79 (N=382)
Hazard ratio
PC1 0.32 (0.063 – 1.6) 0.168
AIC: 809.66; Concordance Index: 0.54
PC2 0.87 (0.18 – 4.1) 0.857
AIC: 811.41; Concordance Index: 0.51
PC3 2.3 (0.26 – 20) 0.459
AIC: 810.88; Concordance Index: 0.51
PC4 4.6 (0.75 – 28) 0.101
AIC: 809.1; Concordance Index: 0.52
PC5 0.22 (0.031 – 1.5) 0.125
AIC: 809.42; Concordance Index: 0.57
PC6 0.62 (0.086 – 4.4) 0.631
AIC: 811.22; Concordance Index: 0.54
b. Univariate analysis: DFS
Events: 74 (N=382)
Hazard ratio
PC1 0.28 (0.051 – 1.6) 0.148
AIC: 734.37; Concordance Index: 0.54
PC2 0.84 (0.16 – 4.4) 0.834
AIC: 736.29; Concordance Index: 0.57
PC3 8.3 (0.71 – 97) 0.091
AIC: 733.28; Concordance Index: 0.61
PC4 15 (3.3 – 72) <0.001 ***
AIC: 727.35; Concordance Index: 0.61
PC5 1.4 (0.12 – 18) 0.781
AIC: 736.26; Concordance Index: 0.52
PC6 0.24 (0.035 – 1.6) 0.147
AIC: 734.39; Concordance Index: 0.58
c. Multivariate analysis: DFS
Hazard ratio
history of polyps NO (N=211) reference
YES (N=112) 0.73 (0.38 – 1.4) 0.34
PC4 (N=323) 19.80 (3.61 – 108.5) <0.001 ***
age (N=323) 0.64 (0.17 – 2.5) 0.519
mutations (N=323) 1.46 (0.29 – 7.3) 0.648
Events: 54; Global p-value (Log-Rank): 0.030977
AIC: 500.86; Concordance Index: 0.67
0.2 0.5 1 2 5 10 20 50 100 200

Fig S13. (a) Univariate Cox proportional-hazard model of overall survival (OS) on the first six principal components (PCs) of colon adenocarcinoma (COAD) principal component analysis (PCA). (b) Univariate Cox proportional-hazard model of disease-free survival (DFS) on the first six PCs of COAD PCA. (c) Cox proportional-hazard model of DFS on PC4 of COAD PCA and its three associated clinical and molecular properties (age, history of colon polyps and mutation burden). AIC, Akaike's Information Criterion.

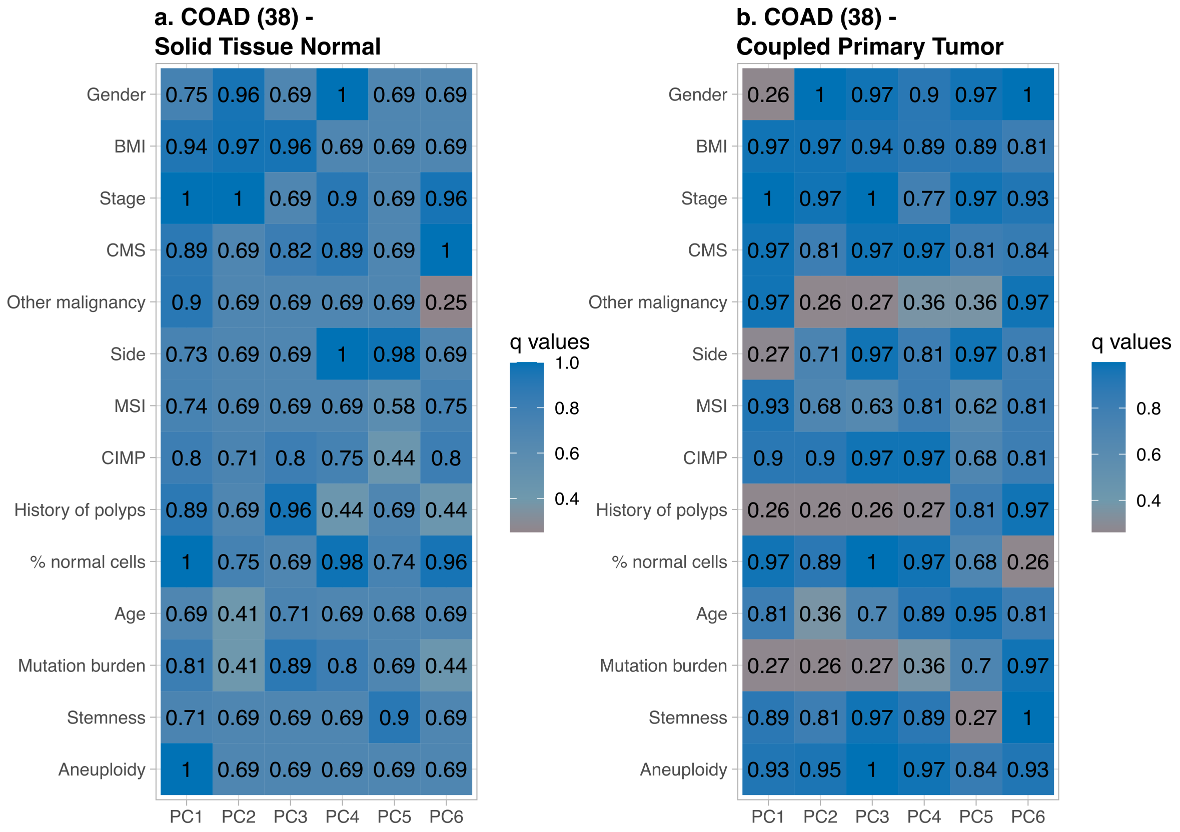

Fig S14. Heatmaps of the *q* values of the associations and correlation between the first six principal components (PCs) of the principal component analysis on the reconstructed microbiome of (a) solid tissue normal colon adenocarcinoma (COAD) samples (non-pathological samples), and the clinical properties from the metadata of each sample (or inherited by their primary tumour coupled samples) and (b) the same analysis on the coupled primary tumour samples. CIMP, CpG methylation phenotype; MSI, microsatellite instability; BMI, body mass index. Number of samples analysed in brackets.

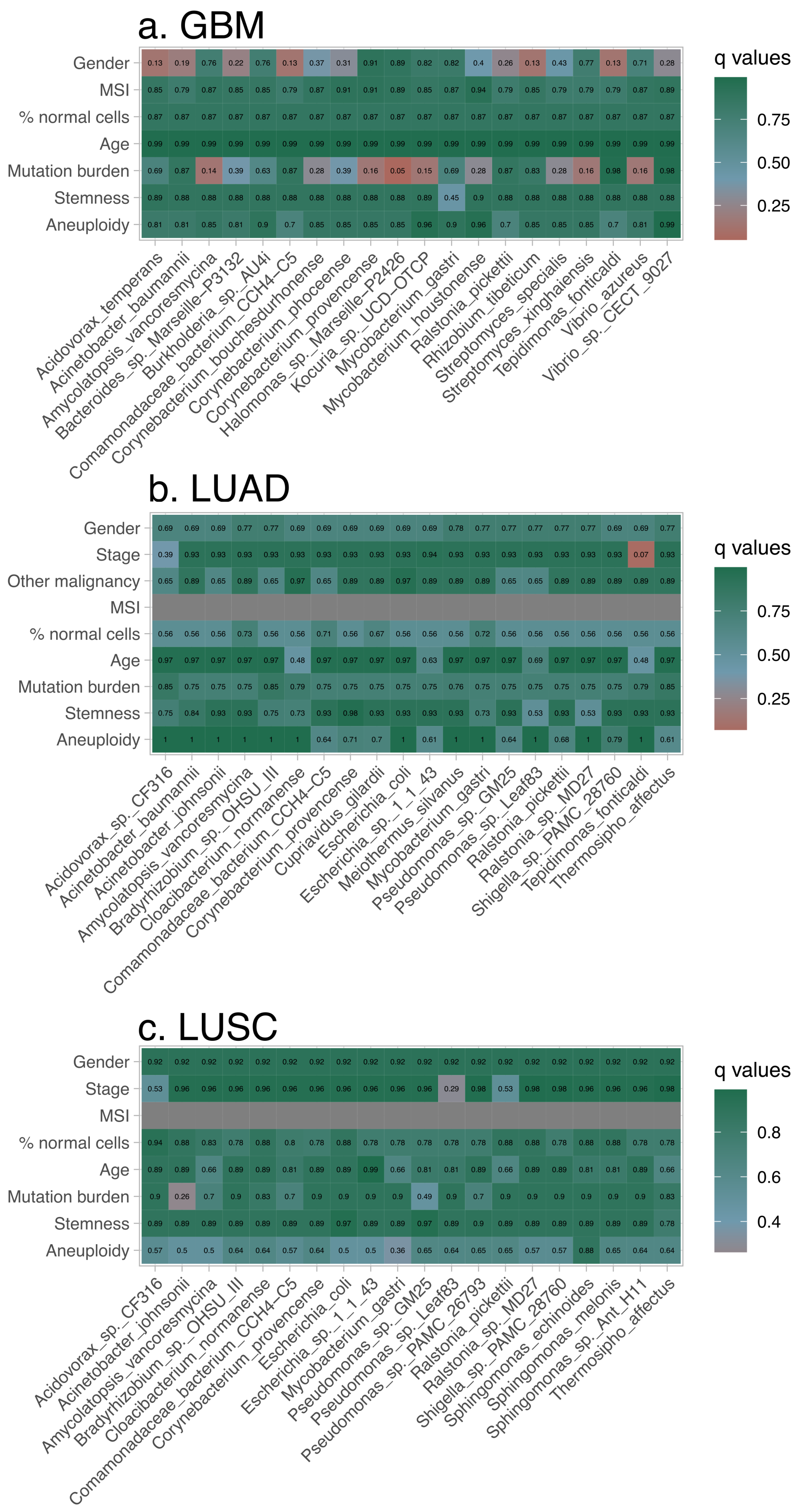
a. GBM
Gender
MSI
% normal cells
Age
Mutation burden
Stemness
Aneuploidy
Acidovorax_temperans
Acinetobacter_baumannii
Amycolatopsis_vancoresmycina
Bacteroides_sp._Marseille-P3132
Burkholderia_sp._AU4i
Comamonadaceae_bacterium_CCH4-C5
Corynebacterium_bouchesdurhonense
Corynebacterium_phoceense
Corynebacterium_provencense
Halomonas_sp._Marseille-P2426
Kocuria_sp._UCD-OTCP
Mycobacterium_gastri
Mycobacterium_houstonense
Ralstonia_pickettii
Rhizobium_tibeticum
Streptomyces_specialis
Streptomyces_xinghaiensis
Tepidimonas_fonticaldi
Vibrio_azureus
Vibrio_sp._CECT_9027
q values
0.75
0.50
0.25
b. LUAD
Gender
Stage
Other malignancy
MSI
% normal cells
Age
Mutation burden
Stemness
Aneuploidy
Acidovorax_sp._CF316
Acinetobacter_baumannii
Acinetobacter_johnsonii
Amycolatopsis_vancoresmycina
Bradyrhizobium_sp._OHSU_III
Cloacibacterium_normanense
Comamonadaceae_bacterium_CCH4-C5
Corynebacterium_provencense
Cupriavidus_gilardii
Escherichia_coli
Escherichia_sp._1_1_43
Meiothermus_silvanus
Mycobacterium_gastri
Pseudomonas_sp._GM25
Pseudomonas_sp._Leaf83
Ralstonia_pickettii
Ralstonia_sp._MD27
Shigella_sp._PAMC_28760
Tepidimonas_fonticaldi
Thermosipho_affectus
q values
0.75
0.50
0.25
c. LUSC
Gender
Stage
MSI
% normal cells
Age
Mutation burden
Stemness
Aneuploidy
Acidovorax_sp._CF316
Acinetobacter_johnsonii
Amycolatopsis_vancoresmycina
Bradyrhizobium_sp._OHSU_III
Cloacibacterium_normanense
Comamonadaceae_bacterium_CCH4-C5
Corynebacterium_provencense
Escherichia_coli
Escherichia_sp._1_1_43
Mycobacterium_gastri
Pseudomonas_sp._GM25
Pseudomonas_sp._Leaf83
Pseudomonas_sp._PAMC_26793
Ralstonia_pickettii
Ralstonia_sp._MD27
Shigella_sp._PAMC_28760
Sphingomonas_echinoides
Sphingomonas_melonis
Sphingomonas_sp._Ant_H11
Thermosipho_affectus
q values
0.8
0.6
0.4

d. HNSC

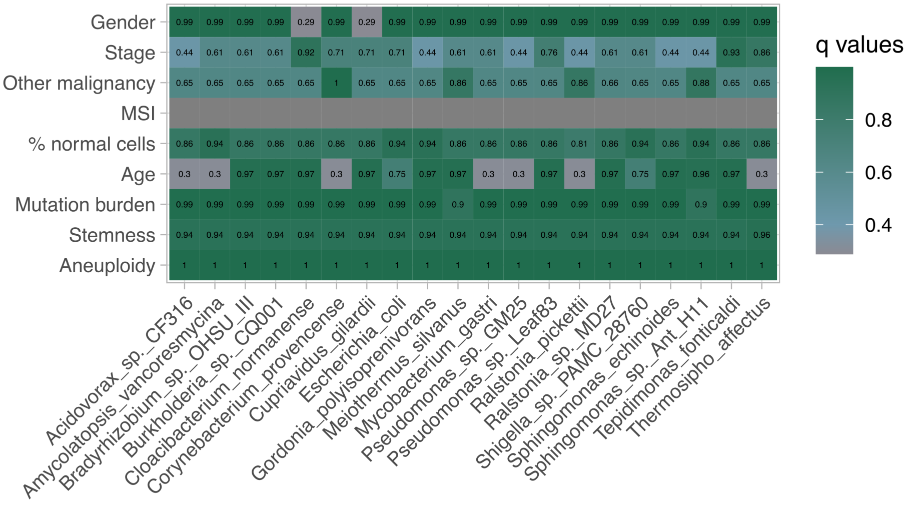

e. OV

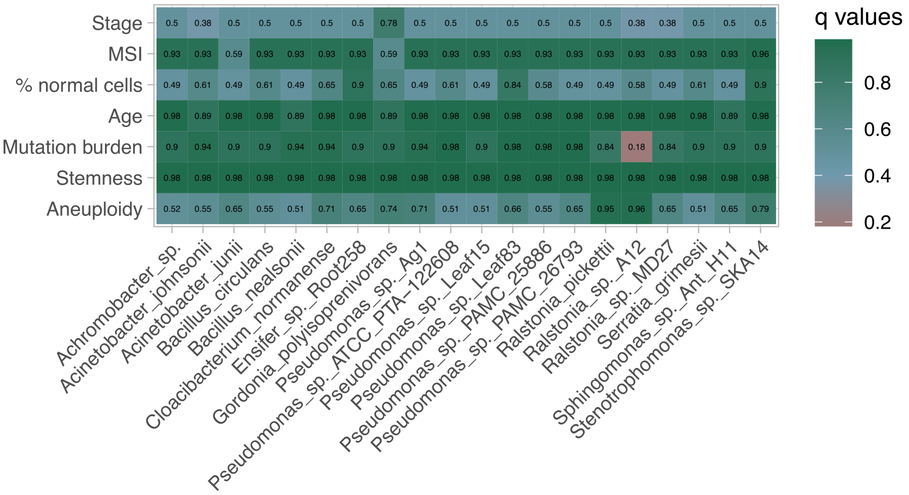

f.READ

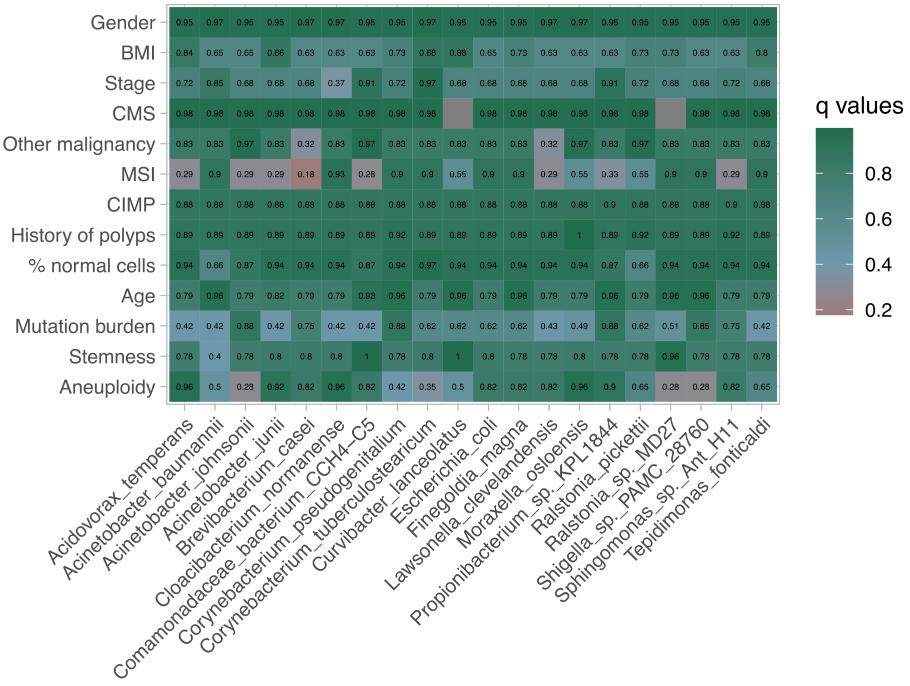

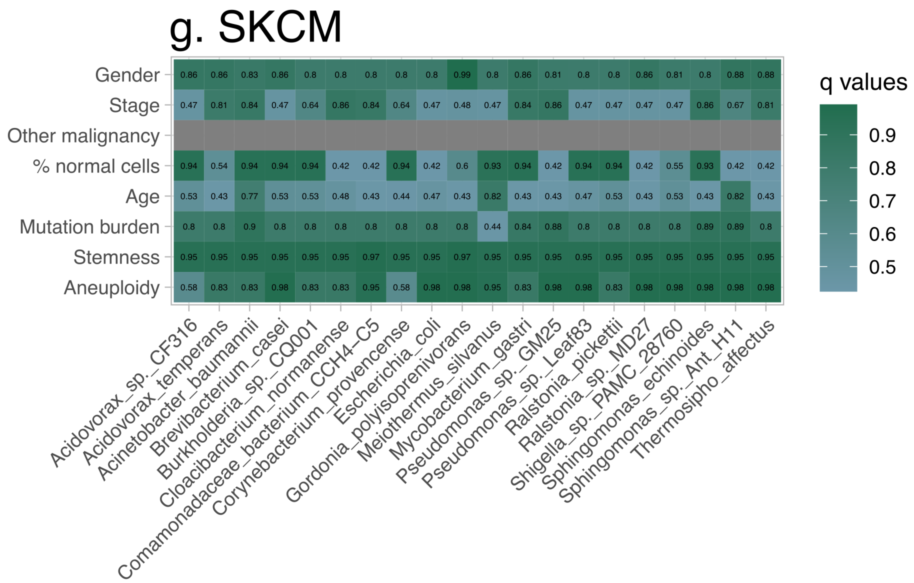

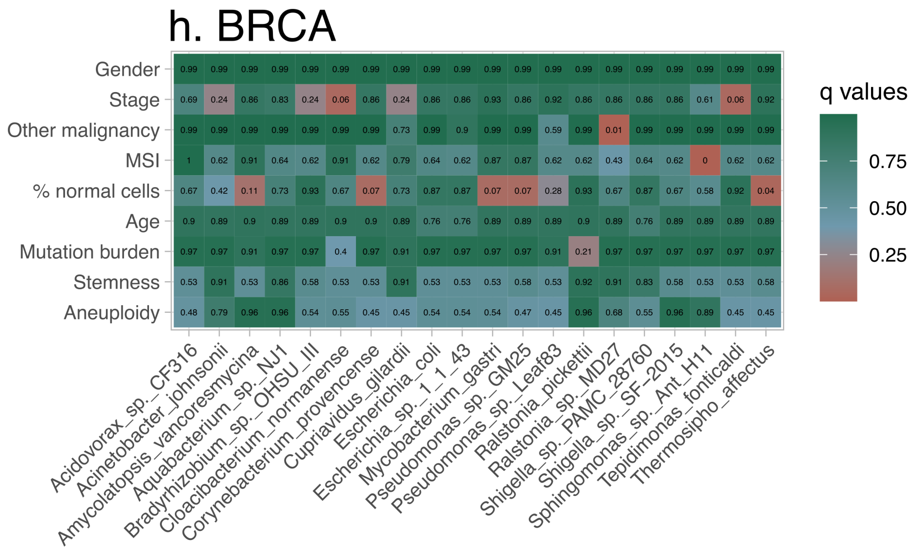

Fig S15. Heatmaps of the *q* values of the associations and correlations between the most prevalent bacterial species (prevalence > 10% of the samples) with the clinical properties of (a) glioblastoma multiforme (GBM), (b) lung adenocarcinoma (LUAD), (c) lung squamous cell carcinoma (LUSC), (d) head and neck squamous cell neoplasms (HNSC), (e) ovarian serous cystadenocarcinoma (OV), (f) rectum adenocarcinoma (READ), (g) skin cutaneous melanoma (SKCM) and (h) breast invasive carcinoma (BRCA). CIMP, CpG methylation phenotype; MSI, microsatellite instability; BMI, body mass index; CMS, consensus molecular subtype.

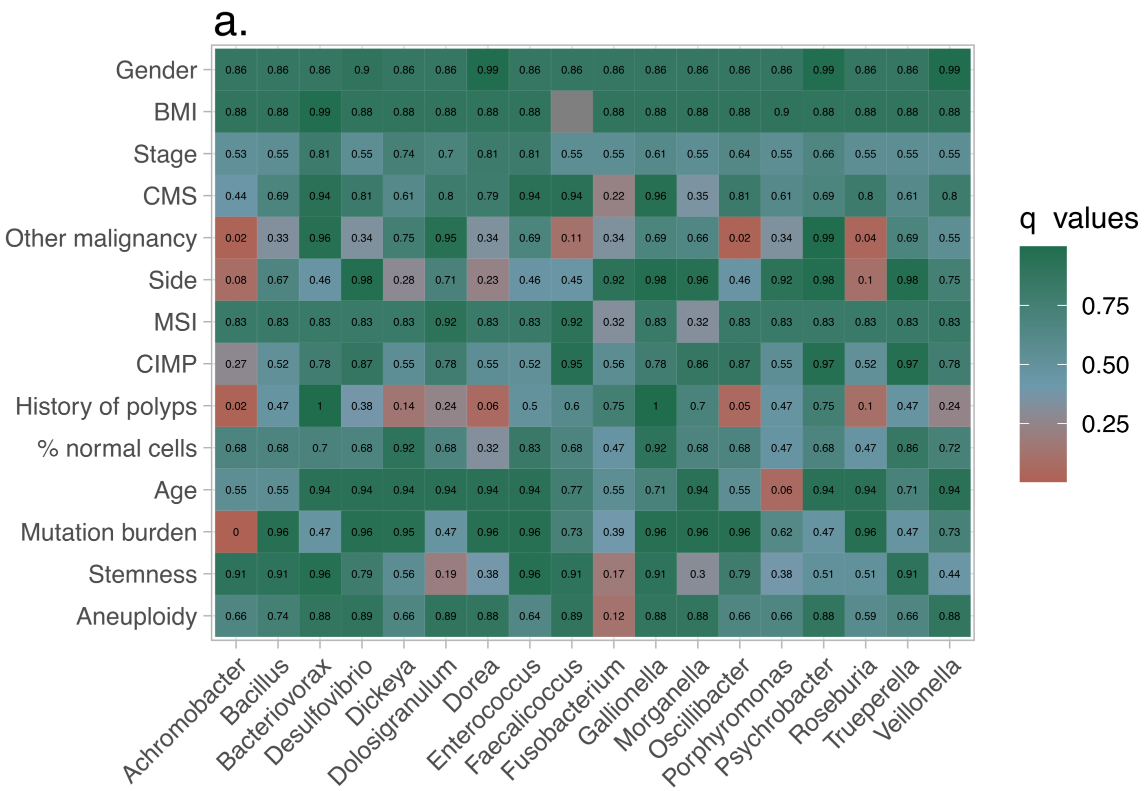

Fig S16. Heatmaps of the *q* values of the associations and correlation between the most reliable bacterial genera (Spearman R > 0.25 in RNA-Seq vs. 16S comparison of European Institute of Oncology cohort) with the clinical properties of colon adenocarcinoma. CIMP, CpG methylation phenotype; MSI, microsatellite instability; CMS, consensus molecular subtype; BMI, body mass index.
